# Exploring Self-Management–Based Mobile Health User Typologies and Associations Between User Types and Satisfaction With Key Mobile Health Functions: Comparative Study of Various Fitness and Weight Management App User Types

**DOI:** 10.2196/64860

**Published:** 2026-02-10

**Authors:** Tong Wang, Yiwen Fan, Zheng Li, Xiaoyi Jiao, Qichuan Fang, Junhao Ma, Jianbo Lei

**Affiliations:** 1 Department of Cardiology and Institute of Vascular Medicine, Peking University Third Hospital; State Key Laboratory of Vascular Homeostasis and Remodeling Peking University Beijing China; 2 University of Health and Rehabilitation Sciences Qingdao Hospital (Qingdao Municipal Hospital), School of Health and Life Sciences University of Health and Rehabilitation Sciences Qingdao, Shandong China; 3 Department of Oncology The Affiliated Hospital of Southwest Medical University Luzhou China; 4 Department of Rehabilitation Medicine & Physical Therapy The Affiliated Hospital of Southwest Medical University Luzhou China; 5 Center for Health Policy Studies School of Public Health Zhejiang University Hangzhou China; 6 Department of AI and IT, Second Affiliated Hospital, School of Medicine Zhejiang University Hangzhou, Zhejiang China; 7 School of Medical Technology and Information Engineering Zhejiang Chinese Medical University Hangzhou China; 8 School of Public Health Hangzhou Medical College Hangzhou China; 9 Institute of Advanced Clinical Medicine Center for Medical Informatics Peking University Beijing China; 10 Clinical Research Center Affiliated Hospital of Southwest Medical University Luzhou China; 11 The First Affiliated Hospital Hainan Medical University Haikou China

**Keywords:** mHealth app, user satisfaction, self-management characteristics, user profile, unsupervised learning

## Abstract

**Background:**

Exploring user satisfaction is crucial for enhancing and ensuring the sustainable development of mobile health (mHealth) apps, particularly in the fitness and weight management sectors. Analyzing user types and developing user profiles are valuable for understanding differences in satisfaction. However, prior research lacks a classification of user types based on self-management characteristics and an analysis of satisfaction disparities among these types.

**Objective:**

This study analyzes user heterogeneity from a self-management perspective among fitness and weight management app users by identifying user types and constructing profiles. It further explores differences in satisfaction with the functional design of these mHealth apps across user types.

**Methods:**

First, 8 feature indicators were selected based on the Health Belief Model and the Behavior Change Wheel to evaluate users’ levels of health knowledge and beliefs, as well as self-regulation related to self-management. Existing research was integrated to categorize mHealth app functional design into 5 categories: health guidance, health education, health monitoring, social features, and gamification. Second, a questionnaire survey was used to collect data on users’ 8 health management characteristics and their satisfaction with the 5 functional design categories. A total of 2518 responses were collected, of which 1025 were included in the analysis. Cluster analysis was conducted to classify users into distinct types based on the 8 health management characteristics, and user profiles were constructed according to the distribution of these characteristics within each type. Finally, the Kruskal-Wallis test was used to analyze differences in satisfaction across user types with respect to the 5 functional design categories of mHealth apps.

**Results:**

Cluster analysis revealed that users could be categorized into 6 types based on the 8 self-management characteristics: positively proactive energizers, proactive intenders, negatively proactive energizers, low health management demanders, potential health management demanders, and passive attitude holders. Significant differences were observed across all 8 health management characteristics among the 6 user types (all *P*<.001). The Kruskal-Wallis test indicated significant variations in user satisfaction with the 5 functional designs of mHealth apps: H(4)=445.388, (*P*<.001). Overall, users reported the highest satisfaction with health guidance and health monitoring (median 4.00, IQR 1.00) and the lowest satisfaction with gamification (median 3.00, IQR 1.00). Positively proactive energizers, proactive intenders, and negatively proactive energizers demonstrated the highest satisfaction with health education and health guidance (median 4.00). Potential health management demanders, proactive intenders, positively proactive energizers, and negatively proactive energizers reported the highest satisfaction with health monitoring (median 4.00). Proactive intenders reported the highest satisfaction with social features and gamification (median 4.00).

**Conclusions:**

Users of mHealth apps exhibit diverse types, with significant differences in health management characteristics and satisfaction with the 5 functional designs of fitness and weight management apps. This study clarifies individual-level differences in user satisfaction with mHealth apps.

## Introduction

The poor continued use of mobile health (mHealth) apps poses a significant threat to the sustainability of the mHealth industry, while also weakening the benefits of health management for users. Approximately 40% of smartphone users utilize mHealth apps [1]. Research indicates that health management based on mHealth apps often requires continued use for over 3 weeks to demonstrate effective outcomes [2]. However, a real-world observational study revealed that only 2.58% of the 189,770 users who downloaded mHealth apps actively used them for at least one week [3], indicating that continued use of mHealth faces serious challenges. Furthermore, app uninstallations result in average monthly losses of US $57,000 for app companies [4], highlighting the negative impact of poor continued use on the industry. Therefore, it is necessary to research the usability of mHealth. Previous studies have shown that user satisfaction is a key determinant of acceptance and continued use [5].

User satisfaction is an important indicator for measuring the quality of mHealth services and has a significant impact on the continued usage and intention to use mHealth [6]. Exploring the factors related to user satisfaction has important reference value for promoting the use of mHealth. A review of existing research on mHealth satisfaction shows that studies mainly follow 3 perspectives: technology acceptance, functional design, and user characteristics. Research from the perspective of technology acceptance is primarily based on the technology acceptance model and its derivative theories, focusing on how users’ perceived characteristics of mHealth technology influence satisfaction [7-11]. Studies on the influencing factors of satisfaction have gradually expanded from a practical dimension to multidimensional value [12], including perceived entertainment and other nonpractical characteristics [13]. Research from the perspective of functional design mainly focuses on how specific functional characteristics of mHealth meet user needs and improve satisfaction, such as data analysis, monitoring, and information sharing [14,15]. This line of research shows a trend of transition from overall evaluation to modular analysis, with the research focus shifting from overall satisfaction with mHealth to the exploration of multidimensional satisfaction [16-18].

Current research has conducted relatively in-depth explorations of mHealth satisfaction from the perspectives of technology acceptance and functional design; however, evidence regarding the relationship between heterogeneity in user characteristics and satisfaction remains limited [19]. A survey on the use of digital health tools among the US population revealed that there is no “one-size-fits-all” model in digital health, as consumers are not a homogeneous group [20]. However, digital health users cannot be classified solely based on sociodemographic characteristics, as each generation interacts with technology differently due to its unique experiences, needs, and levels of trust in the health care system [21]. Previous studies have confirmed that eHealth literacy [22], strong self-reporting capabilities [19], health needs [23], and poor health status [24] significantly influence mHealth satisfaction. Intrinsic characteristics, such as health knowledge, beliefs, and self-regulation skills and abilities, are central to self-management [25]. These findings suggest that self-management attributes provide important explanations for user heterogeneity and differences in satisfaction among users. Compared with traditional health care models, mHealth relies more heavily on self-management service models [26]. Systematic exploration of individual self-management–related characteristics can help construct user classifications that more deeply capture internal features, thereby promoting personalized and refined mHealth design [27]. However, there is a lack of research systematically integrating self-management characteristics and analyzing user-group heterogeneity based on combinations of these characteristics. In addition, empirical evidence on the differential distribution of satisfaction across different user types with respect to mHealth function design remains scarce, posing challenges for developers seeking to deliver mHealth services that meet the needs of diverse users.

To address the above limitations, this study, guided by self-management theory, analyzes relevant self-management characteristics based on the Health Belief Model (HBM) and Behavior Change Wheel (BCW) among users of fitness and weight management apps. A refined classification of these mHealth users is conducted based on characteristic attributes, and differences in satisfaction with mHealth functional design across user types are examined. Through this investigation, the study seeks to address the following research questions:

- Research question 1: User typology: How can users of fitness and weight management apps be systematically categorized using quantitative methods based on self-management characteristics? What are the defining attributes of each user type?
- Research question 2: Satisfaction differences. Do user groups with distinct self-management characteristics demonstrate statistically significant differences in satisfaction with various mHealth functional designs? What are the specific manifestations of these differences, and which factors are relevant?

## Methods

### Design

**Figure 1:**
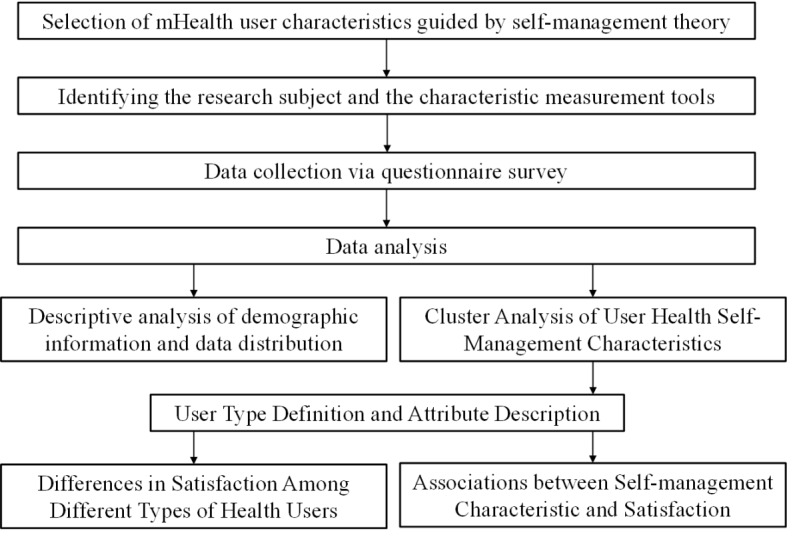
Study flowchart. mHealth: mobile health.

Guided by self-management theory, this study utilizes the HBM and BCW to select variables representing the self-management characteristics of mHealth users. Data were collected through a questionnaire survey, and unsupervised clustering analysis was used to identify user clusters. Subsequently, the characteristic distributions of different user clusters were analyzed to construct user profiles. Through intergroup difference analysis, this study compared satisfaction levels across user types with respect to various mHealth functional designs and examined the correlations between user characteristics and satisfaction with functional designs. The overall workflow of the study is illustrated in Figure 1.

### Selection of Self-Management–Related Characteristics

mHealth mainly improves health outcomes by empowering self-management abilities [28]. Therefore, this study selects variables representing user characteristics guided by self-management theory. In individual and family self-management theory [29], self-management refers to the process by which individuals and families use knowledge and beliefs, self-regulation skills and abilities, and social facilitation strategies to achieve health-related outcomes in their real-life environments. Among these components, knowledge and beliefs refer to information and perceptions related to a health condition or health behavior. The HBM is one of the most widely used models for explaining beliefs and perceptions underlying health behaviors [30]. HBM posits that individuals’ perceptions of their susceptibility to a health issue and the potential severity of its consequences, together with perceived benefits of adhering to health recommendations and their intention and ability to engage in health management, are the most direct drivers of health behaviors [31]. HBM operationalizes these factors through perceived susceptibility, perceived severity, perceived benefits, perceived barriers, and self-efficacy [32]. Accordingly, this study adopts these 5 variables from HBM to characterize the knowledge and beliefs component of self-management theory.

Self-regulation within self-management theory is an iterative process in which individuals utilize a variety of skills and abilities to achieve healthy behavior change [29]. The BCW, a classic theoretical model for explaining behavior change—particularly in the contexts of health promotion and disease prevention [33]—provides an important reference for selecting self-regulation–related characteristic variables in this study. The BCW posits that healthy behavior change can be achieved only when individuals possess capability, motivation, and opportunity [34]. Capability refers to the knowledge and skills required to engage in a particular behavior [35]. In the context of using mHealth for health management, essential knowledge and skills are reflected in 2 aspects: health management and the use of digital technology. eHealth literacy is among the most commonly used indicators for assessing an individual’s ability to acquire health management information and utilize electronic health information to address health issues [36,37]. Accordingly, this study selects eHealth literacy to characterize self-regulation skills and abilities. In addition, motivation in the BCW refers to the beliefs that trigger behavioral change, encompassing reflective motivation and intrinsic motivation [34]. Motivation for health management can be divided into proactive health management intention and passive health management intention triggered by perceived health status, corresponding to reflective motivation and intrinsic motivation, respectively. Therefore, this study selects health management intention [38] and perceived health status [39] to further characterize the motivational dimension of self-management behavior. In summary, this study selects a total of 8 variables to analyze self-management characteristics among mHealth users. The definitions of each variable are presented in Table 1.

**Table 1:** mHealth^a^ user self-management characteristics: a framework of 8 variables for user categorization.

User self-management characteristics	Definition
Perceived susceptibility	Beliefs about the likelihood of getting a disease or condition [31].
Perceived severity	Feelings about the seriousness of contracting an illness or of leaving it untreated, including evaluations of both medical and clinical consequences and possible social consequences [31].
Perceived benefits	Beliefs that engaging in health behaviors can help decrease the risk of diseases and health problems [40].
Perceived barriers	An individual’s assessment of the difficulties and cost of adopting behaviors [32].
Health self-efficacy	The confidence individuals have in their ability to use their skills to complete health management tasks [41].
eHealth literacy	The ability to seek, find, evaluate, and use electronic health information from electronic resources to address health issues [36].
Perceived health status	The perception of one’s own health status [39].
Health management intention	The degree of intention to engage in health protection [38].

^a^mHealth: mobile health.

### Identification of Study Targets

The main purpose of this study is to delineate individuals’ health management characteristics and classify users based on their levels of health management characteristics, and to further analyze differences in satisfaction with various mHealth app functional designs across user categories. Therefore, it is necessary to clarify the main functional designs of mHealth apps. Previous studies have summarized common mHealth app functional designs through systematic reviews and content analyses [42-44], categorizing functions according to their primary implementation purposes, such as education, tracking, social interaction, gamification, and motivational features [42]. Building on prior research, this study reorganizes the functional characteristics reported in the literature to propose a classification of mHealth app functional designs for analysis (see Table 2).

**Table 2:** Five functional designs and features of the mHealth^a^ apps analyzed in this study

Functional designs	Definition	Features
Health guidance	Providing advice based on health plans and applications may provide real-time feedback and advice based on health plans and status [45], or offer strategies for behavior change that can contribute to improving health conditions [46].	Intelligent virtual assistants and communication with health care personnel [43]; interaction with medical staff [45]; and development of health plans and goal setting [46].
Health education	Offering necessary knowledge to achieve health behavior change [47].	Personalized education and general knowledge education [42]; image-based education, video education, and audio education [47].
Health monitoring	Recording past and current health statuses [47].	Health records and health diaries [43]; trackers, data entry, and data export [42]; and self-tracking [46].
Social function	Providing a platform for user interaction, communication, and maintaining connections [47].	Community forums and social media [42]; and social sharing [47].
Gamification	Integrating gaming mechanisms or elements into mHealth apps and enhancing the user experience with gamelike features [48].	Personalized avatars, challenges, tasks, health, and rewards [43]; points, badges, and levels [42].

^a^mHealth: mobile health.

To achieve the aforementioned research objectives, this study selected appropriate objects of study for empirical analysis. First, the comparability of different mHealth app functional designs was considered. To minimize the influence of differences in core service content across app types on comparability, this study selected a homogeneous category of apps as the empirical focus, thereby reducing potential confounding effects. Second, the application domains of different mHealth app categories were considered. Exercise, fitness, and weight management align with substantial health needs. These domains represent important modalities for primary prevention, and exercise therapy also serves as a key approach for tertiary prevention (disease management) in various conditions, such as cardiac rehabilitation, pulmonary rehabilitation, tumor rehabilitation, and metabolic disease management [49]. Given that exercise, fitness, and weight management apps account for more than half of all mHealth apps [50], and that these functions represent 48.37% of users’ motivations for using mHealth apps [51], we selected exercise, fitness, and weight management apps as the units of analysis to represent a broader and more heterogeneous user population.

This study did not restrict respondents to users of a specific app for the following reasons. First, as the aim of this study is to examine the satisfaction of different user types with various mHealth functional designs in real-world contexts and to provide generalizable recommendations, the focus is on mHealth functional modules rather than on specific app design implementations. Accordingly, the research sample was not limited to users of a single app. Moreover, this questionnaire-based data collection approach captures multiple implementations of the same functional module (eg, health education), thereby enhancing sample diversity and improving the generalizability of the findings [52,53]. Second, this study was conducted in real-world settings, which differ from laboratory-based studies that examine factors influencing mHealth satisfaction. In laboratory environments, variables must be tightly controlled to quantify the idealized effects of specific factors, often requiring the use of a single app as the research object. By contrast, this study emphasizes user satisfaction with mHealth in real-world contexts, where the use of multiple similar apps by the same user is common. Analyzing such data provides real-world evidence that can inform future research and practical mHealth design.

### Measurement Instrument

Based on the constructed User Health Management Characteristics Framework, this study measures 8 independent variables and 5 dependent variables reflecting satisfaction with mHealth functionalities. To achieve the aforementioned research objectives, it was necessary to select appropriate and measurable items corresponding to the characteristics defined within the framework. To ensure the rational design of the measurement scale, this study undertook a careful process of adapting previously validated scales, making nuanced adjustments to align them with the specific research objectives.

Specifically, the User Health Management Characteristics Framework includes 4 variables derived from the HBM (perceived severity, perceived susceptibility, perceived barriers, and perceived benefits), with measurement items adapted from HBM-related studies by Ahadzadeh et al [54], Saghafi-Asl et al [32], and McArthur et al [55]. Two variables were derived from the BCW (eHealth literacy and perceived health status), with measurement items drawn from BCW-related studies [39,56,57] and the Brief Health Literacy Screen scale [58]. In addition, 2 variables integrating both models (health self-efficacy and health management intention) were included, with measurement items adapted from health management–related studies, such as Zhou et al [59] and Li et al [38]. For the measurement of satisfaction with the 5 mHealth functional designs, this study first provided definitions and specific design examples of each function in the questionnaire, followed by item-based measurement. The satisfaction items were adapted from relevant studies on mHealth satisfaction [8]. All measurement items used a 5-point Likert scale, ranging from 1 (strongly disagree) to 5 (strongly agree).

A multistage validation and refinement process was implemented to ensure the conceptual accuracy and cultural appropriateness of the English-originated scales within the Chinese context. First, the preliminary translation and adaptation of the scale items were collaboratively conducted by research team members proficient in both Chinese and English and with expertise in health informatics. Through group discussions, each item was refined to preserve the core meaning of the original construct while aligning with Chinese linguistic norms and the research context. Second, before the formal survey, 24 participants with backgrounds in medical informatics were invited to complete a pretest of the questionnaire. Participants were asked not only to respond to the questionnaire but also to provide feedback on the clarity and contextual appropriateness of the items. Based on this feedback, the wording of the questionnaire was further refined to eliminate ambiguities and improve readability, resulting in the final version used in the formal survey. Finally, during the data analysis phase, reliability and validity tests were conducted on the measurement model, thereby verifying the effectiveness and reliability of the measurement instrument from a data-driven perspective (see Multimedia Appendix 1).

### Data Collection

The survey targeted individuals who had used exercise, fitness, or weight loss apps within the preceding 3 months. According to the N:q hypothesis [60], the ratio of sample size to item parameters in a scale should range from 20:1 to 10:1. In this study, a total of 51 items were included for measurement; therefore, applying the more conservative ratio of 20:1, the required sample size was approximately 1020.

From June 28, 2022, to September 8, 2022, a snowball sampling survey was conducted via the WeChat platform (Tencent Holdings Limited). The questionnaire link, generated using Wenjuanxing, was distributed through Moments (Tencent Holdings Limited) and various group chats. In total, 2518 questionnaires were collected. Of these, 902 respondents reported that they had not used the target apps within the preceding 3 months. A quality control item was included in the questionnaire: “This question is an attention test to ensure careful completion of the questionnaire. For this question, please select ‘strongly disagree’.” The correct response to this item was “1. strongly disagree.” Responses that answered this item incorrectly were considered to have failed the quality control check. In addition, given the length of the questionnaire, responses completed in less than 3 minutes or with identical answers across all scale items were deemed insufficiently attentive and were also excluded. Through this screening process, 591 responses were removed. Consequently, 1025 valid questionnaires remained for subsequent analysis. Before completing the questionnaire, all participants were informed of the study’s overall purpose and detailed procedures.

In this study, item-level missing data were addressed using mode imputation based on each respondent’s answers to other items within the same variable. Specifically, there were 3 missing values in total: 1 in perceived susceptibility, 1 in eHealth literacy, and 1 in gamification. As the questionnaire employed Likert scales, which represent ordinal rather than strictly continuous data, each missing value was replaced with the mode of the remaining items within the same scale for that respondent. The imputed values were 3, 5, and 3, respectively. Given that the dataset comprised a total of 45,100 item responses, the proportion of missing values was 0.0067%, which is negligible and unlikely to affect the results.

### Data Analysis

To address the question “What are the common combinations of health management characteristics in users during the process of using mHealth?” we employed cluster analysis. Cluster analysis is an unsupervised machine learning technique that divides a set of observations into multiple clusters composed of similar objects and can identify the structural composition of different clusters. In clustering, similarity between observations within the same cluster is high, whereas similarity between observations from different clusters is low. Among various clustering methods, the k-means algorithm produces results that are easy to interpret, are suitable for clustering high-dimensional data, and demonstrate good scalability and efficiency when dealing with large datasets [61]. The clustering in this study was based on 8 health management characteristics (Table 1), and the sample size was moderate; therefore, k-means was appropriate for this study. The k-means algorithm requires the number of clusters to be predetermined. We determined the optimal number of clusters by integrating the Elbow Method and the Silhouette Coefficient. The Elbow Method identifies the inflection point by analyzing the relationship between the within-cluster sum of squared errors (SSE) and the number of clusters, while the Silhouette Coefficient is used to mathematically assess the compactness and separation of the clustering structure. The results showed that when the number of clusters was 6, an inflection point appeared in the SSE curve, and the Silhouette Coefficient reached a locally optimal value of 0.1514 (see Multimedia Appendix 2), indicating that this clustering solution maintains good within-cluster compactness while achieving favorable between-cluster separation. Therefore, we ultimately set the number of clusters to 6. To further verify the stability of this 6-cluster solution, we performed 10 repeated experiments with random initializations. The results showed a relative fluctuation rate of only 0.002% in the SSE values, demonstrating that the 6-cluster solution exhibits high repeatability and stability. We conducted the cluster analysis to determine the grouping of mHealth app users using sklearn.cluster in Python 3.8 (Python Foundation; see Multimedia Appendix 3).

To answer the question “Is there a difference in satisfaction with the functional design of mHealth apps among different types of users?” we performed rank sum tests to examine satisfaction and preferences regarding functionalities across user groups using SPSS 26 (IBM Corp). To address the question “How do health management characteristics affect users’ satisfaction and preferences for the functional design of mHealth apps?” we conducted a partial least squares analysis using SmartPLS 4 (SmartPLS GmbH). The significance level was set at *P*<.05 for 2-tailed tests.

The data collected for this study were analyzed to assess potential nonresponse bias. To accomplish this and examine nonresponse bias [11], the Mann-Whitney *U* test was used to compare responses from the first 20% (205/1025) and the last 20% (205/1025) of the sample. The results showed no significant differences between the 2 groups for the health guidance (*z*=−0.728, *P*=.47), health education (*z*=−1.171, *P*=.24), health monitoring (*z*=−0.857, *P*=.39), social function (*z*=−1.794, *P*=.07), and gamification (*z*=−0.260, *P*=.80) constructs. Therefore, nonresponse bias was not a concern in this study. This study followed the STROBE (Strengthening the Reporting of Observational Studies in Epidemiology) reporting standard (see Multimedia Appendix 4).

### Ethical Considerations

This study utilized an online questionnaire survey. On the first page of the questionnaire, the research purpose, content, potential risks, data usage, confidentiality terms, and researcher contact information were clearly explained to participants. By checking a box, participants confirmed that they had read and understood the above information and voluntarily agreed to participate in the study, thereby providing informed consent. Participants who did not agree with the above content were directed to exit the online questionnaire. The questionnaire materials for this study were destroyed after the completion of quality control and transcription, within 6 months after the survey concluded. Any associated information, such as source IP addresses, collection time stamps, and serial numbers, was also destroyed to ensure privacy protection. Following transcription, the data were renumbered sequentially, starting from 1. All images and data presented in the manuscript or multimedia appendices are fully anonymized and cannot be used to identify any individual participant or user. No financial compensation was provided to participants. Approval was obtained from the Biomedical Ethics Committee of the Second Affiliated Hospital, School of Medicine, Zhejiang University, China (approval number IRB-2021-841).

## Results

### Demographic Information

The demographic characteristics of the included sample are presented in Table 3. The sample exhibited a balanced gender distribution (female: 553/1025, 53.95%; male: 472/1025, 46.05%), with participants predominantly aged 19-30 years (500/1025, 48.78%). In terms of education, most respondents held college degrees (519/1025, 50.63%), followed by master’s degrees (269/1025, 26.24%). Income distribution showed that nearly one-quarter of participants (244/1025, 23.80%) earned ≤2500 RMB (1 RMB=US $0.14) monthly, whereas a smaller proportion (49/1025, 4.78%) reported incomes of 50,001 or higher. Regarding usage patterns, the largest group reported using the service 2-3 times per week (370/1025, 36.10%), while a minority were frequent users (>7 times per week: 59/1025, 5.76%). In terms of occupation, students constituted the largest subgroup (283/1025, 27.61%), followed by public servants/clerks (208/1025, 20.29%) and professional technical personnel (196/1025, 19.12%). Overall, 664 of 1025 (64.78%) users reported using 1 app.

**Table 3:** Descriptive statistics of demographic information for the analyzed sample (n=1025).

Characteristics			Values, n (%)
**Gender**			
	Male	472 (46.05)	
	Female	553 (53.95)	
**Age (years)**			
	≤18	6 (0.59)	
	19-30	500 (48.78)	
	31-45	337 (32.88)	
	46-60	174 (16.98)	
	≥61	8 (0.78)	
**Education**			
	High school	37 (3.61)	
	Junior college education	111 (10.83)	
	College	519 (50.63)	
	Master’s degree	269 (26.24)	
	Doctor’s degree	89 (8.68)	
**Income (RMB^a^)**			
	≤2500	244 (23.80)	
	2501-5000	159 (15.51)	
	5001-8000	178 (17.37)	
	8001-15,000	213 (20.78)	
	15,001-30,000	125 (12.20)	
	30,001-50,000	57 (5.56)	
	≥50,001	49 (4.78)	
**Frequency**			
	≤1 time per week	265 (25.85)	
	2-3 times per week	370 (36.10)	
	4-5 times per week	213 (20.78)	
	6-7 times per week	118 (11.51)	
	>7 times per week	59 (5.76)	
**Occupation**			
	Students	283 (27.61)	
	Medicine-related personnel	168 (16.39)	
	Public servants and clerks	208 (20.29)	
	Commercial and service personnel	107 (10.44)	
	Professional technical personnel	196 (19.12)	
	Manual workers	33 (3.22)	
	Unemployed and other personnel	30 (2.93)	

^a^1 RMB=US $0.14.

### Data Characteristics

A descriptive analysis of the 8 self-management characteristics and satisfaction with the 5 functional designs was conducted. First, we calculated the average score of multiple items measuring each characteristic variable to represent the level of that variable. However, because the distributions of the average values for each variable were not normally distributed, medians and IQRs were used for presentation, as shown in Table 4.

**Table 4:** Descriptive statistics (median and IQR) of the 8 health management characteristics and satisfaction with the 5 functional designs for the analyzed sample (n=1025).

Characteristics		Median	First quartile (Q1)	Third quartile (Q3)	IQR
**Health-specific determinants**					
	Perceived severity	4.25	3.75	5.00	1.25
	Perceived susceptibility	3.50	3.00	4.00	1.00
	Perceived barrier	2.50	2.00	3.50	1.50
	Perceived benefit	4.75	4.00	5.00	1.00
	Health self-efficacy	4.00	3.33	4.33	1.00
	Perceived health status	3.67	3.00	4.00	1.00
	eHealth literacy	4.00	3.50	4.50	1.00
	Health management intention	4.00	3.33	4.33	1.00
**Satisfaction**					
	Satisfaction with health guidance	4.00	3.00	4.00	1.00
	Satisfaction with health education	3.67	3.00	4.00	1.00
	Satisfaction with health monitoring	4.00	3.33	4.33	1.00
	Satisfaction with social function	3.33	3.00	4.00	1.00
	Satisfaction with gamification	3.00	3.00	4.00	1.00

### Cluster Analysis of User Self-Management Characteristics and Determination of User Types

**Figure 2:**
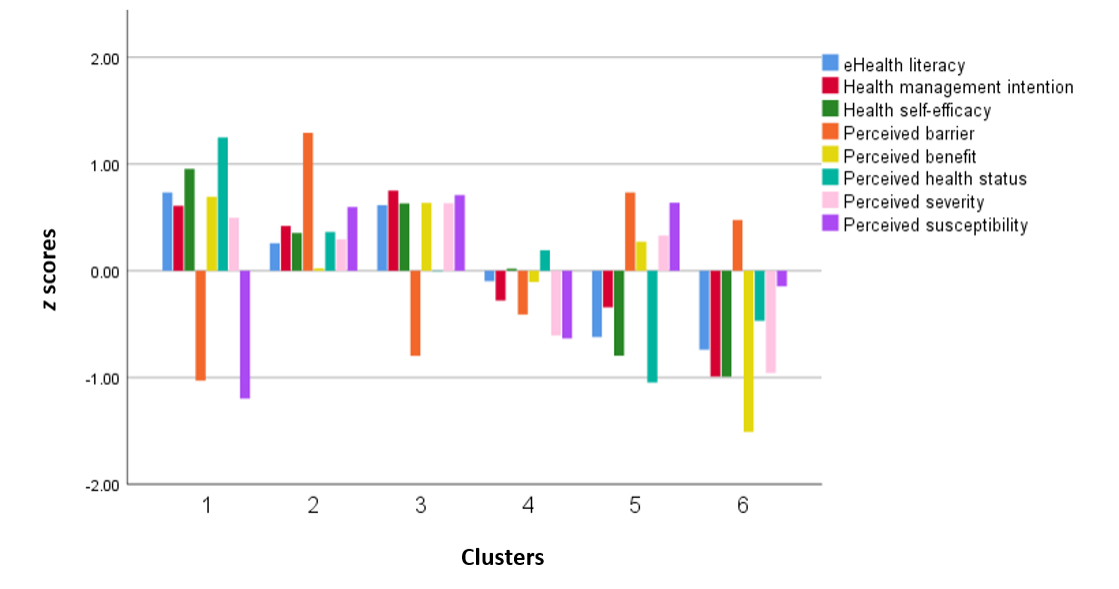
Cluster analysis–derived profiles of 6 user groups across 8 health management characteristics (*z* scores).

The determination of the optimal number of clusters in this study, which was set at 6, was based on the relationship between the within-cluster SSE and the number of clusters. Subsequently, cluster analysis was performed on the sample using *z* score–standardized values of the 8 health management indicators. The distribution across the 6 clusters was relatively uniform (140, 135, 189, 218, 180, and 163). Figure 2 shows the distribution of each indicator across the 6 clusters obtained from the analysis. A *z* score close to 0 indicates that the corresponding indicator in a given cluster approximates the overall average level, whereas a positive *z* score indicates that the indicator is above the average level in that cluster.

We further analyzed differences in each indicator across the 6 clusters using the Kruskal-Wallis test to assess the distinctiveness of each cluster. The results of the Kruskal-Wallis test demonstrated statistically significant differences in the *z* scores of all indicators across the 6 clusters (*P*<.001 for all). Specifically, significant differences were observed for the following: perceived severity, *H*(5)=400.947 (*P*<.001); perceived susceptibility, *H*(5)=522.425, (*P*<.001); perceived barrier, *H*(5)=673.985, (*P*<.001); perceived benefit, *H*(5)=492.630, (*P*<.001;) health self-efficacy, *H*(5)=518.298, (*P*<.001); perceived health status, *H*(5)=490.411, (*P*<.001); eHealth literacy, *H*(5)=362.910, (*P*<.001); and health management intention, *H*(5)=390.573, (*P*<.001). These findings indicate that the 6 clusters exhibit distinct patterns and magnitudes of health management characteristics, as reflected by the significant differences in the *z* scores of the respective indicators. The detailed *z* scores and pairwise comparison results for each indicator across the 6 clusters are presented in Table 5, allowing for a detailed examination of how each cluster differs from the others in terms of specific health management characteristics. Detailed test statistics are provided in Multimedia Appendix 5, which presents pairwise comparisons of health management characteristics among user groups.

The distribution of the 8 health management traits varied across individual users. To further understand user health management characteristics and provide references for personalized services in mHealth apps, this study categorized users based on combinations of their 8 current health management factors. Given the distinctiveness of each user group, the 6 types were named according to the indicator profiles within each cluster to reflect the main health management characteristics of users in each type. Cluster 1 was labeled “positively proactive energizers.” Users in this cluster exhibited higher perceived health status, health management self-efficacy, and health management intention than proactive intenders, along with substantially lower perceived barriers and perceived susceptibility.

Cluster 2 was labeled “proactive intenders,” characterized by users whose perceived health status, eHealth literacy, and health management intention are all above average, but who also report higher perceived barriers and perceived susceptibility. Proactive intenders (135/1025, 13.17%), the smallest group, represent users who demonstrate strong proactivity in health management, recognize their potential risk for health problems, and actively manage their health despite the presence of perceived barriers.

Cluster 3 was labeled “negatively proactive energizers.” Compared with positively proactive energizers, users in this cluster have slightly below-average perceived health status and higher perceived susceptibility, yet they exhibit higher health management intention, health management self-efficacy, and eHealth literacy. Both positively proactive energizers (140/1025, 13.66%) and negatively proactive energizers (189/1025, 18.44%) represent groups with high levels of health management cognition, literacy, and capability, along with strong health management intention, making them the primary beneficiaries of current mHealth apps. The key difference between these 2 groups lies in perceived health status, which is higher among positively proactive energizers and below average among negatively proactive energizers. Cluster 4 was labeled “low health management demanders.” Users in this cluster tend to have average perceived health status, with perceived susceptibility, perceived severity, and health management intention all below the average level. Low health management demanders constitute the largest proportion of users (218/1025, 21.27%) and represent a group with relatively good current health, limited emphasis on health management, insufficient awareness of health risks and potential harm, and consequently lower intention to engage in health management.

Cluster 5 was labeled “potential health management demanders.” Users in this cluster have below-average perceived health status and, although their perceived benefits, perceived susceptibility, and perceived severity are above average, they exhibit lower health management intention and higher perceived barriers. Potential health management demanders (180/1025, 17.56%) represent individuals with poor current health status who are aware of the harms associated with health risks and recognize the benefits of health management for improving their condition, yet face substantial barriers to engaging in health management and therefore have a lower intention to do so. This group has both a clear recognition of the importance of health management and an objective need for it due to their poor health status.

Cluster 6 was labeled “passive attitude holders,” comprising users whose perceived health status is below average and who report very low perceived benefits, health management self-efficacy, perceived severity, and health management intention. Passive attitude holders (163/1025, 15.90%) share similarities with low health management demanders, including low perceived benefits, health management self-efficacy, perceived severity, and intention for health management. However, they differ in that passive attitude holders perceive their health status as below average and face higher perceived barriers. This group perceives a decline in health status but lacks the willingness to improve it, placing them at high risk for illness.

From the perspective of age distribution, the 6 user categories exhibited distinct age-structure characteristics. Positively proactive energizers (n=140) comprised 48 (34.3%) aged 19-30 years, 48 (34.3%) aged 31-45 years, 42 (30.0%) aged 46-60 years, and 2 (1.4%) aged ≥61. Proactive intenders (n=135) consisted of 71 (52.6%) aged 19-30 years, 50 (37.0%) aged 31-45 years, 12 (8.9%) aged 46-60 years, and 2 (1.5%) aged ≥61. Negatively proactive energizers (n=189) included 2 (1.1%) aged ≤18, 65 (34.4%) aged 19-30 years, 79 (41.8%) aged 31-45 years, 41 (21.7%) aged 46-60 years, and 2 (1.1%) aged ≥61. Low health management demanders (n=218) comprised 1 (0.5%) aged ≤18, 103 (47.2%) aged 19-30 years, 69 (31.7%) aged 31-45 years, 44 (20.2%) aged 46-60 years, and 1 (0.5%) aged ≥61. Potential health management demanders (n=180) included 2 (1.1%) aged ≤18, 97 (53.9%) aged 19-30 years, 54 (30.0%) aged 31-45 years, 26 (14.4%) aged 46-60 years, and 1 (0.6%) aged ≥61. Passive attitude holders (n=163) were distributed as 1 (0.6%) aged ≤18, 116 (71.2%) aged 19-30 years, 37 (22.7%) aged 31-45 years, and 9 (5.5%) aged 46-60 years. Chi-square tests indicated that the associations between user clusters and both age (Cramer *V*=0.145) and education (Cramer *V*=0.116) groups were weak.

**Table 5:** Mean z scores and Kruskal-Wallis group comparisons of the 8 health management characteristics across the 6 user clusters.

Clustering variables	Cluster 1: positively proactive energizers (n=140)	Cluster 2: proactive intenders (n=135)	Cluster 3: negatively proactive energizers (n=189)	Cluster 4: low health management demanders (n=218)	Cluster 5: potential health management demanders (n=180)	Cluster 6: passive attitude holders (n=163)
Perceived severity	0.496^a,b,c^	0.294^b,e^	0.632^c^	–0.607^d^	0.331^a,e^	–0.956^f^
Perceived susceptibility	–1.197^a^	0.598^b,c,e^	0.709^c,e^	–0.634^d^	0.638^e^	–0.146^f^
Perceived barrier	–1.029^a,c^	1.292^b^	–0.796^c^	–0.410^d^	0.733^e,f^	0.476^f^
Perceived benefit	0.693^a,c^	0.022^b,d^	0.636^c^	–0.106^d^	0.273^e^	–1.510^f^
Health self-efficacy	0.955^a^	0.354^b,c^	0.630^c^	0.020^d^	–0.795^e,f^	–0.993^f^
Perceived health status	1.249^a^	0.363^b,d^	–0.004^c,d^	0.192^d^	–1.047^e^	–0.469^f^
eHealth literacy	0.733^a,c^	0.258^b^	0.615^c^	–0.097^d^	–0.621^e,f^	–0.740^f^
Health management intention	0.608^a,b,c^	0.419^b^	0.751^c^	–0.278^d,e^	–0.342^e^	–0.990^f^

^a-f^In the same row of data, if the same characteristic across the 6 clusters shares the same superscript (eg, “b”), it indicates that the difference between them is not statistically significant. Conversely, if the same characteristic across the 6 clusters does not share the same superscript, it suggests that the difference between them is statistically significant. For instance, cluster 1’s perceived barrier is denoted as –1.029^a,c^, and cluster 3’s perceived barrier is denoted as –0.796^c^; the presence of the superscript “c” in both indicates that the Kruskal-Wallis test for the *z* scores of perceived barrier between cluster 1 and cluster 3 is not statistically significant. Conversely, cluster 2’s perceived barrier is denoted as 1.292^b^. The absence of a common superscript implies that the Kruskal-Wallis test for the *z* scores of perceived barrier between cluster 1 and cluster 2 is statistically significant.

### Differences in Satisfaction With mHealth App Functional Design Among Different Types of Health Users

First, the Kruskal-Wallis test was used to analyze differences in satisfaction with the 5 functional designs of the mHealth app among all users. The Kruskal-Wallis test indicated significant variations in user satisfaction with the 5 functional designs of mHealth apps—*H*(4)=445.388, *P*<.001. Specifically, the median satisfaction levels for the 5 functional dimensions among all users are presented in Table 6. Among these dimensions, health monitoring and health guidance exhibited the highest levels of satisfaction. Detailed satisfaction scores for the 5 functional designs, as well as pairwise comparisons of satisfaction among the 6 clusters, are also presented in Table 6. These results allow for a detailed examination of how each cluster differs from the others in terms of satisfaction with specific functional designs. Detailed test statistics are provided in Multimedia Appendix 6, which presents pairwise comparisons of satisfaction with the 5 functions across user groups.

Specifically, satisfaction with health guidance was higher in clusters 1, 2, 3, and 5 than in cluster 6. Pairwise comparisons revealed that clusters 1 (*z*=6.246, Bonferroni-adjusted *P*<.001), 2 (*z*=8.404, Bonferroni-adjusted *P*<.001), 3 (*z*=6.887, Bonferroni-adjusted *P*<.001), and 5 (*z*=3.674, Bonferroni-adjusted *P*=.004) differed significantly from cluster 6. Satisfaction with health education and health monitoring was significantly higher in clusters 1–3 than in clusters 4–6, with all 9 cross-group pairwise comparisons for each domain yielding statistically significant differences (health education: *z*=2.77-7.95, Bonferroni-adjusted *P*<.001 to .02 and health monitoring: *z*=3.05-7.88, Bonferroni-adjusted *P*<.001 to .03). With respect to social functions and gamification, users in cluster 2 reported higher satisfaction than those in the other 5 clusters. Pairwise comparisons revealed that cluster 2 differed significantly from cluster 1 (*z*=–3.584, Bonferroni-adjusted *P*=.005), cluster 3 (*z*=4.599, Bonferroni-adjusted *P*<.001), cluster 4 (*z*=6.857, Bonferroni-adjusted *P*<.001), cluster 5 (*z*=7.392, Bonferroni-adjusted *P*<.001), and cluster 6 (*z*=5.992, Bonferroni-adjusted *P*<.001) in social function; and from cluster 1 (*z*=–3.999, Bonferroni-adjusted *P*=.001), cluster 3 (*z*=5.015, Bonferroni-adjusted *P*<.001), cluster 4 (*z*=5.880, Bonferroni-adjusted *P*<.001), cluster 5 (*z*=7.200, Bonferroni-adjusted *P*<.001), and cluster 6 (*z*=6.083, Bonferroni-adjusted *P*<.001) in gamification.

**Table 6:** Median satisfaction and Kruskal-Wallis group comparisons regarding the 5 functional designs across the 6 user types.

Dependent variables	Median (IQR)						
	All users (n=1025)	Cluster 1: positively proactive energizers (n=140)	Cluster 2: proactive intenders (n=135)	Cluster 3: negatively proactive energizers (n=189)	Cluster 4: low health management demanders (n=218)	Cluster 5: potential health management demanders (n=180)	Cluster 6: passive attitude holders (n=163)
Satisfaction with health guidance	4.00 (1.00)	4.00^a,b,c,e^ (1.67)	4.00^b,c^ (1.00)	4.00^c^ (1.00)	3.67^d,e,f^ (1.00)	4.00^e^ (1.00)	3.33^f^ (1.00)
Satisfaction with health education	3.67 (1.00)	4.00^a,b,c^ (1.67)	4.00^b,c^ (1.33)	4.00^c^ (1.33)	3.67^d,e^ (1.00)	3.67^e,f^ (1.00)	3.33^f^ (1.00)
Satisfaction with health monitoring	4.00 (1.00)	4.00^a,b,c^ (0.92)	4.00^b,c^ (1.00)	4.00^c^ (1.33)	3.67^d,e,f^ (1.00)	4.00^e^ (0.67)	3.67^f^ (1.00)
Satisfaction with social function	3.33 (1.00)	3.33^a,f,c^ (1.00)	4.00^b^ (1.00)	3.33^c,d,f^ (1.00)	3.00^d,e,f^ (0.67)	3.00^e,f^ (1.00)	3.33^f^ (0.67)
Satisfaction with gamification	3.00 (1.00)	3.00^a,c,d,f^ (1.00)	4.00^b^ (1.33)	3.00^c,d,e,f^ (1.33)	3.00^d,e,f^ (0.67)	3.00^e,f^ (1.33)	3.00^f^ (0.33)

^a^In the same row of data, the group with a superscript “a” is statistically distinct from that with “b,” “c,” “d,” and “e”. However, the group with a superscript “b” is statistically not distinct from that with “b, c” because they share the superscript “b.” Differing grouping labels indicate the heterogeneity across clusters.

### Associations Between User Self-Management Characteristics and Satisfaction With mHealth App Functional Design

In this study, confirmatory factor analysis was used to test the reliability and validity of the questionnaire. The results, as shown in Multimedia Appendix 7, indicate that all constructs had Cronbach α and composite reliability values above 0.7; the average variance extracted for each construct was greater than 0.6; and the factor loadings for all items exceeded 0.7, demonstrating good reliability and convergent validity for all constructs. The heterotrait-monotrait ratio (HTMT) method was used to assess discriminant validity. The commonly accepted criterion for establishing discriminant validity is an HTMT value below 0.85. The results indicate that all construct pairs exhibited HTMT values below 0.85, supporting the discriminant validity of the measurement model. Using 8 health management characteristics as independent variables and satisfaction with 5 feature designs as dependent variables, partial least squares analysis was conducted to examine the impact of health management characteristics on satisfaction with the functional design of mHealth apps. The *R*^2^ value represents the proportion of variance in the dependent variable explained by the independent variables and is used to interpret the goodness-of-fit of the regression equation, while *β* indicates the correlation between variables. Table 7 displays the *β* values and statistical significance of *R*^2^ for the independent and dependent variables. Specifically, perceived barriers (*β*=.177, *z*=3.586, *P*<.001), perceived susceptibility (*β*=.076, *z*=2.056, *P*=.04), health self-efficacy (*β*=.140, *z*=3.307, *P*=.001), perceived health status (*β*=.111, *z*=2.967, *P*=.003), eHealth literacy (*β*=.153, *z*=3.947, *P*<.001), and health management intention (*β*=.148, *z*=3.966, *P*<.001) had a significant positive impact on satisfaction with health guidance; perceived barriers (*β*=0.143, *z*=3.130, *P*=.002), health self-efficacy (*β*=0.147, *z*=3.491, *P*<.001), perceived health status (*β*= 0.120, *z*=3.072, *P*=.002), eHealth literacy (*β*= 0.131, *z*=3.362, *P*=.001), and health management intention (*β*= 0.175, *z*=4.670, *P*<.001) had a significant positive impact on satisfaction with health education; perceived severity (*β*=.118, *z*=3.235, *P*=.001), perceived barriers (*β*=.107, *z*=2.194, *P*=.03), perceived benefits (*β*=.089, *z*=2.428, *P*=.02), perceived health status (*β*=.088, *z*=2.188, *P*=.03), eHealth literacy (*β*=.170, *z*=4.561, *P*<.001), and health management intention (*β*=.083, *z*=2.064, *P*=.04) had a significant positive impact on satisfaction with health monitoring; perceived susceptibility (*β*=.098, *z*=2.314, *P*=.02), perceived barriers (*β*=.150, *z*=3.032, *P*=.002), health self-efficacy (*β*=.195, *z*=4.598, *P*<.001), perceived health status (*β*=.135, *z*=3.391, *P*=.001), eHealth literacy (*β*=.118, *z*=2.805, *P*=.005), and health management intention (*β*=.139, *z*=3.396, *P*=.001) had a significant positive impact on satisfaction with social functions, whereas perceived benefits (*β*=–.210, *z*=5.906, *P*<.001) had a significant negative impact and perceived susceptibility (*β*=.099, *z*=2.200, *P*=.03), perceived barriers (*β*=.139, *z*=3.074, *P*=.002), health self-efficacy (*β*=.137, *z*=3.076, *P*=.002), perceived health status (*β*=.122, *z*=2.834, *P*=.005), eHealth literacy (*β*=.119, *z*=2.771, *P*=.006), and health management intention (*β*=.100, *z*=2.354, *P*=.02) had a significant positive impact on satisfaction with gamification, while perceived benefits (*β*=–.165, *z*=5.011, *P*<.001) had a significant negative impact. These results help explain why different types of users exhibit varying levels of satisfaction with different functional designs. The statistical analysis results for each path are presented in Multimedia Appendix 8.

**Table 7:** Results of partial least squares analyses for 8 health management characteristics on satisfaction of 5 functional designs (β value).

	Satisfaction with health guidance	Satisfaction with health education	Satisfaction with health monitoring	Satisfaction with social function	Satisfaction with gamification
Perceived severity	.058	.050	.118^a^	.034	–.012
Perceived susceptibility	.076^b^	.049	.046	.098^b^	.099^b^
Perceived barrier	.177^c^	.143^a^	.107^b^	.150^a^	.139^a^
Perceived benefit	.048	–.001	.089^b^	–.210^c^	–.165^c^
Health self-efficacy	.140^a^	.147^c^	.070	.195^c^	.137^a^
Perceived health status	.111^a^	.120^a^	.088^b^	.135^a^	.122^a^
eHealth literacy	.153^c^	.131^a^	.170^c^	.118^a^	.119^a^
Health management intention	.148^c^	.175^c^	.083^b^	.139^a^	.100^b^
*R* ^2^	.183	.168	.152	.144	.094

^a^*P*<.01.

^b^*P*<.05.

^c^*P*<.001.

## Discussion

### Principal Findings

Understanding the heterogeneity of mHealth users and analyzing distribution differences in satisfaction with mHealth function design among user groups with distinct characteristics have significant referential value for personalized mHealth design. Therefore, guided by self-management theory and driven by self-management characteristics, this study constructed mHealth user personas encompassing 6 categories, analyzed the main characteristics and attributes of these 6 types of mHealth users, and examined differences in satisfaction with various mHealth function designs across user types. The results show that levels of user self-management characteristics are unevenly distributed and that different combinations of self-management characteristics can explain user heterogeneity. Moreover, a clear correlation exists between user heterogeneity arising from self-management characteristics and satisfaction with specific function designs. The findings of this study mutually confirm and extend existing literature on differences in mHealth intervention effects, user participation motivation, and technology design principles.

### User Profiles of Fitness and Weight Management Apps Based on Health Management Characteristics

This study categorizes mHealth users into 6 distinct groups based on their self-management characteristics, revealing a complex classification derived from self-management–related attributes such as knowledge, beliefs, self-regulation skills and abilities, and motivation. This classification system differs from traditional approaches that rely on broad demographic labels (such as age and education level), offering a more refined and interpretable psychographic user profile. Although the sample in this study is predominantly composed of young-to-middle-aged and highly educated users, comparison with the 50th Statistical Report on China’s Internet Development (2022) [62] shows that while individuals aged 20-49 account for 56.6% of all Chinese internet users, only 28.5% have used online medical services. This indicates that even within the broadly age-eligible internet population, active adopters of digital health services constitute a specific subgroup. Accordingly, this study employed an online snowball sampling method to reach and recruit active users with genuine interest and engagement in mHealth management. The “young-to-middle-aged, well-educated” characteristics of the sample, to some extent, reflect the profile of individuals who pay close attention to such apps. However, chi-square test results show that the associations between user classification and age (Cramer *V*=0.145) and education level (Cramer *V*=0.116) are weak. Furthermore, analysis of the age composition of each user group revealed that every category spans multiple age layers, suggesting that single demographic labels are insufficient to capture the characteristics of the 6 user categories identified in this analysis. The uneven distribution of multiple features across different user categories indicates that there is no complete positive correlation between individuals’ perceived health status and their health management abilities and motivations. The differentiation of users’ characteristic attributes is consistent with relevant theories such as BCWs. Potential health management demanders and passive attitude holders both exhibit low health management intention, but their underlying mechanisms differ: the former perceive susceptibility and severity but face higher perceived barriers, whereas the latter generally lack recognition of the perceived benefits and health self-efficacy of health management. This is consistent with phenomena observed in studies of smoking cessation apps [63], namely that when users remain at the level of understanding health information but fail to convert it into personalized risk perception and action confidence, behavioral intentions are difficult to activate. Based on the results of this study, targeted interventions in mHealth management are needed according to individuals’ motivation status and health perceptions, to facilitate the transformation of different perception characteristics into internal motivation and to address differences in health management behaviors arising from varying motivation statuses [64]. In addition, although negatively proactive energizers perceive an average health status, they exhibit high health management intention and self-efficacy. This finding indicates that perceived health status alone is not the sole determinant of mHealth service use, which is also supported by existing studies [65], showing that patients’ satisfaction with mHealth services may be influenced by individual differences in disease progression, perceived service attitudes, and understanding of diagnostic and treatment outcomes. Correspondingly, passive attitude holders exhibit low levels of perceived health status, perceived benefits, health self-efficacy, and health management intention, which aligns with a previous study [66] identifying lack of motivation and low perceived value as significant barriers to mHealth participation. In general, obtaining a positive mHealth service experience often depends on whether services can be personalized or adaptively adjusted to accommodate different user needs and motivations, and user heterogeneity is strongly correlated with self-management characteristics [67].

### Satisfaction of Various User Types With mHealth App Functionality Design

There are significant differences in satisfaction with mHealth function designs among different user types, providing counterevidence to the “one-size-fits-all” function design strategy and underscoring the necessity of personalized design. This study found that proactive intenders and positively proactive energizers, characterized by high self-efficacy and strong health management intention, reported higher satisfaction with goal-oriented and interactive functions such as health guidance, health education, and health monitoring. This finding is consistent with multiple studies [63,68,69], indicating that users who actively focus on their own well-being demonstrate higher levels of engagement with mHealth. Features such as progress tracking, personalized feedback, and access to educational content are often welcomed and perceived as useful by these users.

Low health management demanders and passive attitude holders report lower satisfaction with most mHealth functions. This finding is consistent with a previous study [70], which indicated that for users with low intrinsic motivation or insufficient perceived value, complex functions or interfaces may act as barriers to use; such users require designs that are concise, intuitive, provide immediate and intuitive feedback, and involve low operational barriers. Notably, prior research has demonstrated a positive correlation between eHealth literacy and mHealth usage [71]. However, our findings offer a contrasting perspective, showing that potential health management demanders, despite having lower-than-average eHealth literacy, report high satisfaction with health guidance and health monitoring functions. This may be because this group’s higher perceived benefits and perceived susceptibility compensate for their lower capability, thereby increasing their interest in tools that enhance health benefits and reduce health risks. This phenomenon may also be partially explained by the Dunning-Kruger effect, whereby users lacking specialized knowledge may be less able to critically evaluate application limitations, leading to inflated satisfaction ratings for mHealth. Individuals with low health literacy have been shown to report levels of confidence about their knowledge comparable to, or even higher than, those with high literacy [72]. Conversely, users with stronger health knowledge reserves tend to use educational materials and support functions in mHealth apps more frequently, thereby enhancing recognition and evaluation of specific functions [73]. The user-centered design concept, combined with the user types constructed in this study, can therefore provide valuable reference for mHealth designs that address the diverse needs of different user groups.

### Recommendations

The findings of this study provide clear implications for the personalized design and practical application of mHealth. mHealth developers should adopt a user-centered design approach and, based on the user types constructed in this study, tailor functional modules accordingly. For positively proactive energizers, further development of data tracking, personalized goal setting, and systematic educational content can be pursued, alongside the appropriate introduction of gamification elements to enhance users’ sense of enjoyment [74]. For potential health management demanders, design efforts should focus on lowering usage barriers, providing clear and step-by-step health guidance and monitoring tools, and strengthening health-related beliefs. For passive attitude holders, the primary objective may be to establish initial engagement and trust through simple designs and light-touch interventions, while incorporating social features to help this group build social support [75], increase awareness of health risks, and enhance beliefs in health management. Furthermore, the study’s results suggest that when evaluating the effectiveness of mHealth functions, user heterogeneity must be considered, as overall average satisfaction may obscure substantial differences among user subgroups.

### Limitations and Future Research

Although this study was carefully designed in terms of methodology and empirical analysis, several limitations should be acknowledged. First, to avoid comparability issues arising from differences in functional design across mHealth apps with different core service content, and to recruit as diverse a user sample as possible, this study selected exercise, fitness, and weight management apps as the objects of study. However, these apps cannot fully represent all types of mHealth apps. Nevertheless, by conducting empirical research on one of the most widely used and numerous categories of mHealth apps, this study verifies that individuals’ self-management characteristics have important referential value for mHealth user classification. The results demonstrate significant differences in satisfaction with various functional designs across user types, and that the effects of self-management characteristics on satisfaction differ across functional design dimensions. These results provide direct empirical reference for the personalized design of fitness and weight loss apps, and the research conclusions may also offer inspiration for user classification–related studies in other types of mHealth apps. Second, although questionnaires are an effective research method, they cannot fully represent all users. Access to feedback data from the entire mHealth user population would enable a more refined classification of user types. At present, limited research on mHealth apps has focused on users’ health cognition and self-management abilities, which constrains the analysis of comprehensive user characteristics from a big data perspective. To mitigate sampling bias, data were collected from 34 regions across China, and the sample size was substantially larger than that of comparable studies, supporting the representativeness of the findings. Third, although empirical results indicate that 8 self-management characteristics significantly influence user satisfaction, these characteristics explain only a limited proportion of the variance in satisfaction with mHealth (*R*^2^=0.094-0.183). Although the identified pathways were statistically significant, a substantial proportion of the variance in satisfaction remains unexplained by these characteristics. Given that the primary aim of this study was to examine whether the effects of heterogeneous user types on satisfaction differ—that is, to construct user profiles based on self-management characteristics and analyze differences in satisfaction across user types—we did not conduct an in-depth examination of other potential determinants of satisfaction (eg, usage context, service quality, and user experience). Future research on factors influencing mHealth satisfaction could incorporate additional predictive variables to improve the explanatory power of the model.

### Conclusions

This study identified 6 distinct mHealth user typologies (fitness and weight management apps) derived from a comprehensive set of self-management characteristics. The results reveal an uneven distribution of multidimensional characteristics across different user categories. This granular classification provides a deeper understanding of user heterogeneity than traditional demographic-based approaches. Overall, the primary contribution of this work is an empirically validated framework that offers a structured approach to understanding mHealth user diversity. The identified user typologies and their differential functional satisfaction profiles yield actionable insights for the personalized design of fitness and weight management apps. Specifically, for health guidance and education features, proactive intenders, positively proactive energizers, and negatively proactive energizers report higher satisfaction. For health monitoring functions, potential health management demanders, proactive intenders, positively proactive energizers, and negatively proactive energizers demonstrate higher satisfaction. For social features and gamification design, proactive intenders exhibit significantly higher satisfaction than the other 5 user types. From a practical perspective, these findings underscore the importance of adapting mHealth features to the distinct self-management needs and preferences of specific user groups. Such adaptation is essential for enhancing user satisfaction, fostering sustained engagement, and ultimately improving the perceived usefulness of digital health solutions.

## Appendix

Multimedia Appendix 1 Measurement instrument.

Multimedia Appendix 2 Cluster sum of squared errors values and silhouette coefficients.

Multimedia Appendix 3 Clustering analysis code.

Multimedia Appendix 4 STROBE checklist.

Multimedia Appendix 5 Comparison of health management characteristics between pairs of user groups.

Multimedia Appendix 6 Comparison of satisfaction across 5 functions between paired user groups.

Multimedia Appendix 7 Reliability and validity analysis.

Multimedia Appendix 8 Path coefficients.

## Abbreviations

BCW: Behavior Change Wheel
HBM: Health Belief Model
HTMT: heterotrait-monotrait ratio
mHealth: mobile health
SSE: sum of squared errors
STROBE: Strengthening the Reporting of Observational Studies in Epidemiology

## Data Availability

The data will be made available upon reasonable request to the corresponding author.

## References

[1] Khalil AA, Hidayanto AN, Prabowo H, Meyliana (2020). Identification of factor affecting continuance usage intention of mHealth application: a systematic literature review.

[2] Wang Y, Xue H, Huang Y, Huang L, Zhang D (2017). A systematic review of application and effectiveness of mHealth interventions for obesity and diabetes treatment and self-management. Adv Nutr.

[3] Helander E, Kaipainen K, Korhonen I, Wansink B (2014). Factors related to sustained use of a free mobile app for dietary self-monitoring with photography and peer feedback: retrospective cohort study. J Med Internet Res.

[4] AppsFlyer (2020). The uninstall ThreatApp uninstall benchmarks. AppsFlyer.

[5] Wang T, Wang W, Liang J, Nuo M, Wen Q, Wei W, Han H, Lei J (2022). Identifying major impact factors affecting the continuance intention of mHealth: a systematic review and multi-subgroup meta-analysis. NPJ Digit Med.

[6] Wang T, Zheng X, Liang J, An K, He Y, Nuo M, Wang W, Lei J (2022). Use of machine learning to mine user-generated content from mobile health apps for weight loss to assess factors correlated with user satisfaction. JAMA Netw Open.

[7] Yang M, Al Mamun A, Gao J, Rahman MK, Salameh AA, Alam SS (2024). Predicting m-health acceptance from the perspective of unified theory of acceptance and use of technology. Sci Rep.

[8] Wang T, Fan L, Zheng X, Wang W, Liang J, An K, Ju M, Lei J (2021). The impact of gamification-induced users' feelings on the continued use of mHealth apps: a structural equation model with the self-determination theory approach. J Med Internet Res.

[9] Wang T, Wang W, Feng J, Fan X, Guo J, Lei J (2024). A novel user-generated content-driven and Kano model focused framework to explore the impact mechanism of continuance intention to use mobile apps. Computers in Human Behavior.

[10] Hashemi P, Reychav I, Arora A, Sawaed N, Sabherwal R, Azuri J (2024). How does information technology affect individuals’ health behavior in a pandemic? Insights from mobile health application use during COVID-19. Computers in Human Behavior.

[11] Singh P, Malik G (2024). From trial to triumph! A longitudinal design eliciting social impact perspective for sustained usage of gamified fitness apps. APJML.

[12] Han K, Zo H (2023). Understanding the mobile healthcare applications continuance: the regulatory focus perspective. Int J Med Inform.

[13] Nam M, Song T, Kim D, Jang K, Kim J, Koo B, Lee J, Chung M (2023). Effects of content characteristics and improvement in user satisfaction on the reuse of home fitness application. Sustainability.

[14] He Y, Chen H, Xiang P, Zhao M, Li Y, Liu Y, Wang T, Liang J, Lei J (2024). Establishing an evaluation indicator system for user satisfaction with hypertension management apps: combining user-generated content and analytic hierarchy process. J Med Internet Res.

[15] Nuo M, Zheng S, Wen Q, Fang H, Wang T, Liang J, Han H, Lei J (2023). Mining the influencing factors and their asymmetrical effects of mHealth sleep app user satisfaction from real-world user-generated reviews: content analysis and topic modeling. J Med Internet Res.

[16] Luna-Perejon F, Malwade S, Styliadis C, Civit J, Cascado-Caballero D, Konstantinidis E, Abdul SS, Bamidis PD, Civit A, Li Y-C (2019). Evaluation of user satisfaction and usability of a mobile app for smoking cessation. Comput Methods Programs Biomed.

[17] Lee H, Uhm KE, Cheong IY, Yoo JS, Chung SH, Park YH, Lee JY, Hwang JH (2018). Patient satisfaction with mobile health (mHealth) application for exercise intervention in breast cancer survivors. J Med Syst.

[18] Arensman R, Kloek C, Pisters M, Koppenaal T, Ostelo R, Veenhof C (2022). Patient perspectives on using a smartphone app to support home-based exercise during physical therapy treatment: qualitative study. JMIR Hum Factors.

[19] Fu HN, Adam TJ, Konstan JA, Wolfson JA, Clancy TR, Wyman JF (2019). Influence of patient characteristics and psychological needs on diabetes mobile app usability in adults with type 1 or type 2 diabetes: crossover randomized trial. JMIR Diabetes.

[20] (2025). Screenagers to silver surfers: how each generation clicks with care. Rock Health.

[21] Conway N, Campbell I, Forbes P, Cunningham S, Wake D (2016). mHealth applications for diabetes: user preference and implications for app development. Health Informatics J.

[22] Nie L, Oldenburg B, Cao Y, Ren W (2023). Continuous usage intention of mobile health services: model construction and validation. BMC Health Serv Res.

[23] Lee J, Kim J (2019). Can menstrual health apps selected based on users' needs change health-related factors? A double-blind randomized controlled trial. J Am Med Inform Assoc.

[24] Jamison RN, Mei A, Ross EL (2016). Longitudinal trial of a smartphone pain application for chronic pain patients: predictors of compliance and satisfaction. J Telemed Telecare.

[25] Ryan P, Sawin KJ (2009). The individual and family self-management theory: background and perspectives on context, process, and outcomes. Nurs Outlook.

[26] Zhou Y, Li S, Huang R, Ma H, Wang A, Tang X, Pei R, Piao M (2024). Behavior change techniques used in self-management interventions based on mHealth apps for adults with hypertension: systematic review and meta-analysis of randomized controlled trials. J Med Internet Res.

[27] Leziak K, Birch E, Jackson J, Strohbach A, Niznik C, Yee LM (2021). Identifying mobile health technology experiences and preferences of low-income pregnant women with diabetes. J Diabetes Sci Technol.

[28] Irfan Khan A, Gill A, Cott C, Hans PK, Steele Gray C (2018). mHealth tools for the self-management of patients with multimorbidity in primary care settings: pilot study to explore user experience. JMIR Mhealth Uhealth.

[29] Bauer W, Schiffman R, Ellis J, Erickson J, Polfuss M, Taani M, Sawin K (2025). An integrative review of the use of the individual and family self-management theory in research. Advances in Nursing Science.

[30] Suess C, Maddock J, Dogru T, Mody M, Lee S (2022). Using the Health Belief Model to examine travelers' willingness to vaccinate and support for vaccination requirements prior to travel. Tour Manag.

[31] Glanz K, Rimer B, Viswanath K (2008). Health Behavior and Health Education: Theory, Research, and Practice.

[32] Saghafi-Asl M, Aliasgharzadeh S, Asghari-Jafarabadi M (2020). Factors influencing weight management behavior among college students: an application of the Health Belief Model. PLoS One.

[33] Chauhan BF, Jeyaraman MM, Mann AS, Lys J, Skidmore B, Sibley KM, Abou-Setta AM, Zarychanski R (2017). Behavior change interventions and policies influencing primary healthcare professionals' practice-an overview of reviews. Implement Sci.

[34] Michie S, Atkins L, West R (2014). The Behaviour Change Wheel: A Guide to Designing Interventions.

[35] Michie S, van SMM, West R (2011). The Behaviour Change Wheel: a new method for characterising and designing behaviour change interventions. Implement Sci.

[36] Norman CD, Skinner HA (2006). eHealth literacy: essential skills for consumer health in a networked world. J Med Internet Res.

[37] World Health Organization (WHO) (2021). Health promotion glossary of terms. WHO.

[38] Li W, Yuan K, Yue M, Zhang L, Huang F (2021). Climate change risk perceptions, facilitating conditions and health risk management intentions: evidence from farmers in rural China. Climate Risk Management.

[39] Jeong H, Lee Y (2020). Sex-based differences in the quality of life of elderly Koreans with chronic musculoskeletal pain. Int J Environ Res Public Health.

[40] Du J, Luo C, Shegog R, Bian J, Cunningham RM, Boom JA, Poland GA, Chen Y, Tao C (2020). Use of deep learning to analyze social media discussions about the human papillomavirus vaccine. JAMA Netw Open.

[41] Williams DM, Rhodes RE (2016). The confounded self-efficacy construct: conceptual analysis and recommendations for future research. Health Psychol Rev.

[42] Mendiola MF, Kalnicki M, Lindenauer S (2015). Valuable features in mobile health apps for patients and consumers: content analysis of apps and user ratings. JMIR Mhealth Uhealth.

[43] Gimpel H, Manner-Romberg T, Schmied F, Winkler TJ (2021). Understanding the evaluation of mHealth app features based on a cross-country Kano analysis. Electron Markets.

[44] Yoon S, Goh H, Nadarajan GD, Sung S, Teo I, Lee J, Ong MEH, Graves N, Teo TL (2021). Perceptions of mobile health apps and features to support psychosocial well-being among frontline health care workers involved in the COVID-19 pandemic response: qualitative study. J Med Internet Res.

[45] Wilhide Iii Calvin C, Peeples MM, Anthony Kouyaté Robin C (2016). Evidence-based mHealth chronic disease mobile app intervention design: development of a framework. JMIR Res Protoc.

[46] Agarwal P, Gordon D, Griffith J, Kithulegoda N, Witteman HO, Sacha Bhatia R, Kushniruk AW, Borycki EM, Lamothe L, Springall E, Shaw J (2021). Assessing the quality of mobile applications in chronic disease management: a scoping review. NPJ Digit Med.

[47] Wang Y, Collins WB (2021). Systematic evaluation of mobile fitness apps: apps as the tutor, recorder, game companion, and cheerleader. Telematics and Informatics.

[48] Deterding S, Sicart M, Nacke L, O'Hara K, Dixon D, editors (2011). Gamification: using game design elements in non-gaming contexts.

[49] Lv H, Zeng N, Li M, Sun J, Wu N, Xu M, Chen Q, Zhao X, Chen S, Liu W, Li X, Zhao P, Wintermark M, Hui Y, Li J, Wu S, Wang Z (2024). Association between body mass index and brain health in adults: a 16-year population-based cohort and mendelian randomization study. Health Data Sci.

[50] Serrano KJ, Yu M, Coa KI, Collins LM, Atienza AA (2016). Mining health app data to find more and less successful weight loss subgroups. J Med Internet Res.

[51] Albarrak MM, Zakaria N, Meo SA (2024). The role of mobile health applications (mHealth apps) in reshaping the body weight for better healthcare: a cross-sectional study. Pak J Med Sci.

[52] Yin S, Cai X, Wang Z, Zhang Y, Luo S, Ma J (2022). Impact of gamification elements on user satisfaction in health and fitness applications: a comprehensive approach based on the Kano model. Computers in Human Behavior.

[53] Mitchell Robert, Schuster Lisa, Jin Hyun Seung (2022). Playing alone: can game design elements satisfy user needs in gamified mHealth services?. Health Promot Int.

[54] Ahadzadeh AS, Pahlevan Sharif Saeed, Ong FS, Khong KW (2015). Integrating health belief model and technology acceptance model: an investigation of health-related internet use. J Med Internet Res.

[55] McArthur LH, Riggs A, Uribe F, Spaulding TJ (2018). Health belief model offers opportunities for designing weight management interventions for college students. J Nutr Educ Behav.

[56] Norman CD, Skinner HA (2006). eHEALS: The eHealth Literacy Scale. J Med Internet Res.

[57] Austin JD, Allicock M, Atem F, Lee SC, Fernandez ME, Balasubramanian BA (2020). A structural equation modeling approach to understanding pathways linking survivorship care plans to survivor-level outcomes. J Cancer Surviv.

[58] Wallston KA, Cawthon C, McNaughton CD, Rothman RL, Osborn CY, Kripalani S (2014). Psychometric properties of the brief health literacy screen in clinical practice. J Gen Intern Med.

[59] Zhou C, Zheng W, Tan F, Lai S, Yuan Q (2022). Influence of health promoting lifestyle on health management intentions and behaviors among Chinese residents under the integrated healthcare system. PLoS One.

[60] Jackson DL (2003). Revisiting sample size and number of parameter estimates: some support for the N:q hypothesis. Structural Equation Modeling: A Multidisciplinary Journal.

[61] Lou N (2022). Analysis of the intelligent tourism route planning scheme based on the cluster analysis algorithm. Comput Intell Neurosci.

[62] (2022). The 50th statistical report on China's internet development. China Internet Network Information Center.

[63] Tudor-Sfetea C, Rabee R, Najim M, Amin N, Chadha M, Jain M, Karia K, Kothari V, Patel T, Suseeharan M, Ahmed M, Sherwani Y, Siddiqui S, Lin Y, Eisingerich AB (2018). Evaluation of two mobile health apps in the context of smoking cessation: qualitative study of cognitive behavioral therapy (CBT) versus non-CBT-based digital solutions. JMIR Mhealth Uhealth.

[64] Prochaska JO, DiClemente CC (1983). Stages and processes of self-change of smoking: toward an integrative model of change. Journal of Consulting and Clinical Psychology.

[65] Lu J, Zhao H, Ji Z, Dong Y (2025). Effect of patient satisfaction on the utilization of mHealth services by patients with chronic disease. Digit Health.

[66] Alzghaibi H (2025). Healthcare practitioners' perceptions of mhealth application barriers: challenges to adoption and strategies for enhancing digital health integration. Healthcare (Basel).

[67] Wittmar S, Frankenstein T, Timm V, Frei P, Kurpiers N, Wölwer Stefan, Schäfer Axel Georg Meender (2025). User experience with a personalized mHealth service for physical activity promotion in university students: mixed methods study. JMIR Form Res.

[68] Kowalski L, Finnes A, Koch S, Bujacz A (2024). User engagement with organizational mHealth stress management intervention - a mixed methods study. Internet Interv.

[69] Monachelli R, Davis SW, Barnard A, Longmire M, Docherty JP, Oakley-Girvan I (2024). Designing mHealth apps to incorporate evidence-based techniques for prolonging user engagement. Interact J Med Res.

[70] Santos-Vijande ML, Gómez-Rico M, Molina-Collado A, Davison RM (2022). Building user engagement to mHealth apps from a learning perspective: relationships among functional, emotional and social drivers of user value. Journal of Retailing and Consumer Services.

[71] Shaw George, Castro BA, Gunn LH, Norris K, Thorpe Roland J (2024). The association of eHealth literacy skills and mHealth application use among US adults with obesity: analysis of Health Information National Trends Survey Data. JMIR Mhealth Uhealth.

[72] Canady BE, Larzo M (2023). Overconfidence in managing health concerns: the Dunning-Kruger effect and health literacy. J Clin Psychol Med Settings.

[73] Chua JYX, Choolani M, Chee CYI, Yi H, Lalor JG, Chong YS, Shorey S (2025). A mobile app–based intervention (Parentbot–a digital healthcare assistant) for parents: secondary analysis of a randomized controlled trial. J Med Internet Res.

[74] Lin Y, Tudor-Sfetea C, Siddiqui S, Sherwani Y, Ahmed M, Eisingerich AB (2018). Effective behavioral changes through a digital mHealth app: exploring the impact of hedonic well-being, psychological empowerment and inspiration. JMIR Mhealth Uhealth.

[75] Nuseibeh BZ, Johns SA, Shih PC, Lewis GF, Gowan TM, Jordan EJ (2024). Co-designing the MOSAIC mHealth app with breast cancer survivors: user-centered design approach. JMIR Form Res.

