# Supplementary material for: Exploring Self-Management–Based Mobile Health User Typologies and Associations Between User Types and Satisfaction With Key Mobile Health Functions: Comparative Study of Various Fitness and Weight Management App User Types
Source: JMIR Med Inform. 2026 Feb 10;14:e64860. doi: 10.2196/64860 (PMC12933165; doi:10.2196/64860)
Supplement: Multimedia Appendix 1 [file medinform_v14i1e64860_app1.pdf]

Supplementary Table 1. Measurement instrument

Variables and sources	Version	Items
Perceived severity (Saghafi-Asl(2020), McArthur(2018))	Referenced version (Original items in reference)	Being overweight could make me feel anxious and stressed.<br>Being overweight could make me unhappy and depressed.<br>Have an unfavorable effect on my health in years to come.<br>Increase my risk for diabetes; Increase my risk for heart disease; Increase my risk for high blood pressure; Increase my risk for cancer.
	English version (for measuring in this study)	Being overweight or obesity could make me feel anxious and stressed.<br>Being overweight or obesity could make me unhappy and depressed.<br>Being overweight or obesity could have an adverse effect on my health in years to come.<br>Being overweight or obesity could increase my risk for diabetes, high blood pressure, cancer and other illnesses.
	Chinese version (items used in the questionnaire of this study)	超重或肥胖会让我感到焦虑和压力。<br>超重或肥胖会让我不开心和沮丧。<br>超重或肥胖会在未来几年对我的健康产生不利影响。<br>超重或肥胖会增加我患疾病的风险。
Perceived susceptibility (Ahadzadeh(2015), Saghafi-Asl(2020))	Referenced version (Original items in reference)	I have a higher likelihood of getting chronic diseases.<br>I have a strong possibility of attack or deterioration of chronic disease due to improper daily habits (drinking, smoking, dietary habit, lack of exercise, etc).
	English version (for measuring in this study)	I have a high likelihood of getting overweight or obese.<br>I have a strong possibility of attack or deterioration of overweight or obese due to improper daily habits (drinking, dietary habit, lack of exercise, etc).
	Chinese version (items used in the questionnaire of this study)	我有变得超重或肥胖得趋势。<br>由于不良的日常习惯（饮酒、非健康饮食及缺乏锻炼等），我极有可能变得超重或肥胖。

Perceived barrier (Saghafi-Asl(2020))	Referenced version (Original items in reference)	I don’t know how to plan physical activity into my daily Schedule.<br>I don’t know how to choose low-calorie beverages, foods, or snacks.
	English version (for measuring in this study)	I don’t know how to plan physical activity into my daily schedule.<br>I don’t know how to choose low-calorie beverages, foods, or snacks.
	Chinese version (items used in the questionnaire of this study)	我不知道如何将体育活动计划到我的日常安排中。<br>我不知道如何选择低热量的饮料、食物或零食。
Perceived benefit (Saghafi-Asl(2020))	Referenced version (Original items in reference)	It would benefit me to adopt healthy eating and physical activity habits by Reducing anxiety and stress.<br>It would benefit me to adopt healthy eating and physical activity habits by Make me feel more energetic.<br>It would benefit me to adopt healthy eating and physical activity habits by Increase my chances of having good health now and in the future.<br>It would benefit me to adopt healthy eating and physical activity habits by Improve a symptom or health problem I have now.
	English version (for measuring in this study)	It would benefit me to adopt healthy eating and physical activity habits by Reducing anxiety and stress.<br>It would benefit me to adopt healthy eating and physical activity habits by Make me feel more energetic.<br>It would benefit me to adopt healthy eating and physical activity habits by Increase my chances of having good health now and in the future.<br>It would benefit me to adopt healthy eating and physical activity habits by Improve a symptom or health problem I have now.
	Chinese version (items used in the questionnaire of this study)	养成健康饮食和体育锻炼的习惯有助于减少焦虑和压力。<br>养成健康饮食和体育锻炼的习惯可以让我感觉更有活力。<br>养成健康饮食和体育锻炼的习惯将有助于保持健康的状态。<br>养成健康饮食和体育锻炼的习惯会改善我现在的症状或健康问题。
Health self-efficacy (Saghafi-Asl(2020),	Referenced version (Original items in	I can eat meals in moderate amounts and regularly.<br>I can exercise instead of watching TV in leisure time.

Austin(2020),)	reference)	Overall, how confident are you about your ability to take good care of your health?
	English version (for measuring in this study)	I can eat meals in moderate amounts and regularly.<br>I can exercise instead of watching TV or other recreational activities of meditation in leisure time.<br>Overall, how confident are you about your ability to adopt healthy eating and physical activity?
	Chinese version (items used in the questionnaire of this study)	我可以适量且有规律地进行健康饮食。<br>我可以在闲暇时间锻炼，而不是看电视等其他静止娱乐活动。<br>总体而言，您对自己采取健康饮食和体育锻炼的能力有多大信心？
Perceived health status (Austin(2020), Jeong(2020))	Referenced version (Original items in reference)	Health status compared to one year ago.<br>Health status compared to the same age<br>In general, would you say your health is...
	English version (for measuring in this study)	My weight status compared to one year ago.<br>My weight status compared to the same age.<br>In general, my current weight status is......
	Chinese version (items used in the questionnaire of this study)	与一年前相比，我的体重状况......<br>我的体重状况与同龄人相比......<br>总的来说，我目前的体重状况......
eHealth literacy (Norman(2006))	Referenced version (Original items in reference)	I know how to find helpful health resources on the Internet.<br>I know how to use the Internet to answer my health questions.<br>I know what health resources are available on the Internet.<br>I know where to find helpful health resources on the Internet.<br>I know how to use the health information I find on the Internet to help me.<br>I have the skills I need to evaluate the health resources I find on the Internet.<br>I can tell high quality from low quality health resources on the Internet.<br>I feel confident in using information from the Internet to make health decisions.
	English version (for measuring in this study)	I know how to find helpful health resources on the Internet.<br>I know how to use the Internet to answer my health questions.

		I know what health resources are available on the Internet.<br>I know where to find helpful health resources on the Internet.<br>I know how to use the health information I find on the Internet to help me.<br>I have the skills I need to evaluate the health resources I find on the Internet.<br>I can tell high quality from low quality health resources on the Internet.<br>I feel confident in using information from the Internet to make health decisions.
	Chinese version (items used in the questionnaire of this study)	我知道如何上网查找有用的卫生健康信息。<br>我知道如何利用网络来解答自己的健康问题。<br>我知道从网上可以获取的卫生健康信息有哪些。<br>我知道从网络上哪里可以获取到有用的卫生健康信息。<br>我知道如何利用网络中的卫生健康信息来帮助自己。<br>我具备评价网络中卫生健康信息好坏的技能。<br>我能够区分网络上高质量和低质量的卫生健康信息。<br>我对利用网络信息做出健康相关的决定充满信心。
Health management intention (weight management or fitness) (Zhou et al(2022); Li et al(2021))	Referenced version (Original items in reference)	I will give more efforts to learn the ways of health protection.<br>I will give more time to health protection.<br>I am likely to spend more money on health protection.
	English version (for measuring in this study)	I will give more efforts to learn the ways of weight management or fitness.<br>I will give more time to weight management or fitness.<br>I am likely to spend more money on weight management or fitness.
	Chinese version (items used in the questionnaire of this study)	我会更加努力地学习体重管理或体育锻炼的方法。<br>我会花更多时间进行体重管理或体育锻炼。<br>我可能会在体重管理或体育锻炼上花更多钱。
Satisfaction(wang et al(2021))	Referenced version (Original items in reference)	Overall, I am satisfied with the use of MH.<br>My experience with using MH is quite close to my ideal MHS.<br>I feel contented with using internet banking service.

<table>
<tr>
<td></td>
<td>English version (for measuring in this study)</td>
<td>
Health Guidance: Providing real-time feedback and recommendations based on health plans and status, or offering behavior change strategies to improve health outcomes. Such as smart virtual assistants, communication with healthcare providers, health plan development and goal setting.
Health Education: Providing essential knowledge to achieve health behavior change. Examples: Personalized education, general knowledge education, visual education (e.g., infographics, posters), video courses, audio courses.
Health Monitoring: Recording past and present health status. Examples: health records, health journals; trackers, data entry, data export; self-tracking.
Social Function: Provides a platform to facilitate communication, interaction, and connection between users. Examples: Community forums, social media, and social sharing.
Gamification: The integration of game mechanics or elements into mobile health apps to provide users with a gaming experience. Examples: Personalized avatars, challenges, tasks, health rewards; points, badges, and levels.

Overall, I am satisfied with the use of the following features in the weight management app.
<table>
<tr><td></td><td>Very dissatisfied</td><td>Dissatisfied</td><td>Neutral</td><td>Satisfied</td><td>Very satisfied</td></tr>
<tr><td>Health Guidance</td><td></td><td></td><td></td><td></td><td></td></tr>
<tr><td>Health Education</td><td></td><td></td><td></td><td></td><td></td></tr>
<tr><td>Health Monitoring</td><td></td><td></td><td></td><td></td><td></td></tr>
<tr><td>Social Function</td><td></td><td></td><td></td><td></td><td></td></tr>
<tr><td>Gamification</td><td></td><td></td><td></td><td></td><td></td></tr>
</table>

In the weight management app, the following features are designed to be very close to my ideal health management function during my use.
<table>
<tr><td></td><td>Strongly disagree</td><td>Disagree</td><td>Neutral</td><td>Agree</td><td>Strongly agree</td></tr>
<tr><td>Health Guidance</td><td></td><td></td><td></td><td></td><td></td></tr>
</table>
</td>
</tr>
</table>

Health Education					
Health Monitoring					
Social Function					
Gamification					

I feel good about the services provided by the following features.

	Strongly bad	Bad	Neutral	Good	Strongly good
Health Guidance					
Health Education					
Health Monitoring					
Social Function					
Gamification					

Chinese version (items used in the questionnaire of this study)

健康指导： 根据健康计划和状态提供实时反馈和建议，或提供有助于改善健康状态的行为改变策略。

如：智能虚拟助手、与保健人员沟通；与医护人员沟通；健康计划制定、目标设定。

健康教育： 提供必要的知识来实现健康行为改变。

如：个性化教育、常识教育；图片教育、视频课程、音频课程。

健康监测： 记录过去和当前的健康状态。

如：健康记录、健康日记；跟踪器、数据录入、数据导出；自我跟踪。

社交功能： 提供平台实现用户间的交流与互动并保持联系。

如：社区论坛、社交媒体；社交分享。

游戏化： 将游戏机制或元素整合到移动健康 app 中，赋予用户游戏体验。

如：个性化头像、挑战、任务、健康奖励；积分、徽章、等级。

总的来说，我对体重管理 app 中以下功能的使用感到满意。

	非常不满意	不满意	一般	满意	非常满意

健康指导					
健康教育					
健康监测					
社交功能					
游戏化					

体重管理 app 中，以下功能设计在我使用过程中，非常接近我理想的健康管理功能。

	非常不同意	不同意	一般	同意	非常同意
健康指导					
健康教育					
健康监测					
社交功能					
游戏化					

我对以下功能提供的服务感觉良好。

	非常不好	不好	一般	良好	非常好
健康指导					
健康教育					
健康监测					
社交功能					
游戏化					
