# Supplementary material for: Exploring Self-Management–Based Mobile Health User Typologies and Associations Between User Types and Satisfaction With Key Mobile Health Functions: Comparative Study of Various Fitness and Weight Management App User Types
Source: JMIR Med Inform. 2026 Feb 10;14:e64860. doi: 10.2196/64860 (PMC12933165; doi:10.2196/64860)
Supplement: Multimedia Appendix 2 [file medinform_v14i1e64860_app2.pdf]

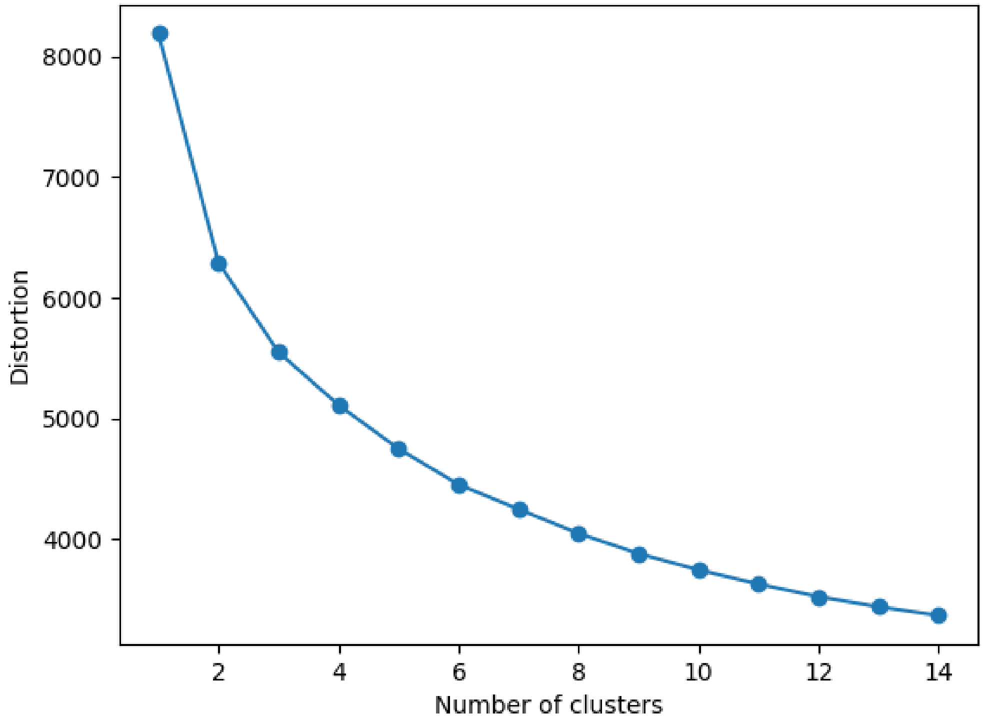

Multimedia Appendix 2. Figure 1. Clusters SSE values

(Range Between 2 and 14 Clusters)

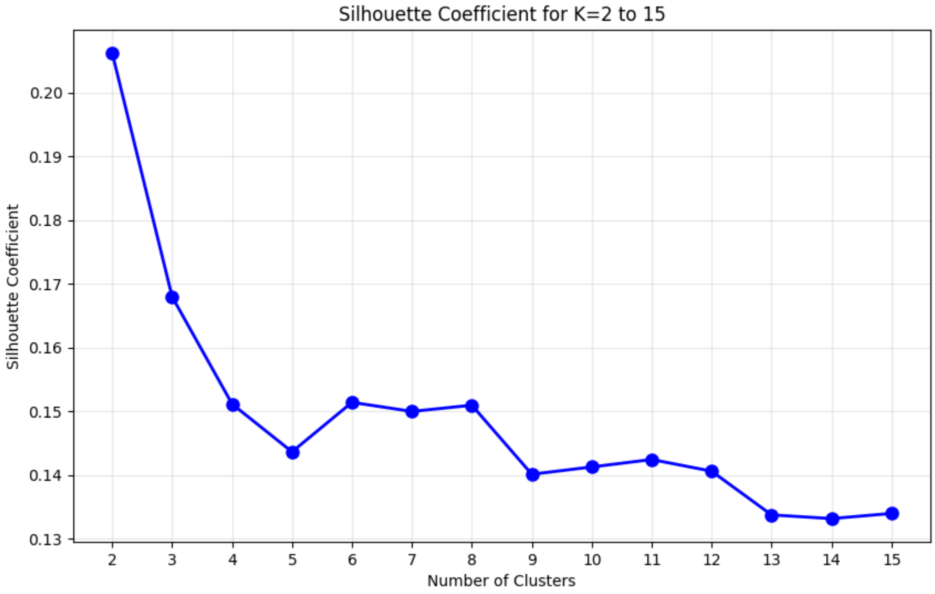

Multimedia Appendix 2. Figure 2. Clusters Silhouette Coefficient value (Range Between 2 and 15 Clusters)
