# Supplementary material for: Exploring Self-Management–Based Mobile Health User Typologies and Associations Between User Types and Satisfaction With Key Mobile Health Functions: Comparative Study of Various Fitness and Weight Management App User Types
Source: JMIR Med Inform. 2026 Feb 10;14:e64860. doi: 10.2196/64860 (PMC12933165; doi:10.2196/64860)
Supplement: Multimedia Appendix 3 [file medinform_v14i1e64860_app3.pdf]

Code related to cluster analysis

```
import pandas as pd
from sklearn.datasets import make_blobs
from sklearn.cluster import KMeans
import matplotlib.pyplot as plt
import numpy as np

data = pd.read_excel("data.xlsx")
x = data.iloc[:, -8:].values
t = list(data.columns[-8:])
np.set_printoptions(suppress=True)

# 1. Main cluster analysis
est = KMeans(n_clusters=6,init='k-means++',n_init=50,max_iter=300)
est.fit(x) # Calculate k-means clustering
kc = est.cluster_centers_ # Calculate centroids
y_kmeans = est.predict(x) # Assign each sample to its closest cluster
appraise = est.inertia_ # Compute the sum of squared distances to cluster centroids
data['cluster'] = est.labels_ # Generate cluster labels
feature = est.n_features_in_ # Number of features seen during fitting
# 2. Stability Validation
print("\n=== Stability Validation ===")
inertia_values = []
for i in range(10):
    est_test = KMeans(n_clusters=6, init='k-means++', n_init=50, max_iter=300,
                      random_state=i)
    est_test.fit(x)
    inertia_values.append(est_test.inertia_)
print("Inertia values from 10 runs:", inertia_values)
print("Standard deviation:", np.std(inertia_values))
print("Relative fluctuation rate: {:.4%}".format(np.std(inertia_values) / np.mean(inertia_values)))

# 3. Output Main Results
print(y_kmeans,kc,'\n')
print(kc.shape,y_kmeans.shape,np.shape,'\n')
print(appraise)

data.to_excel('results.xlsx', index=1) #Append cluster labels
data_kc = pd.DataFrame(kc, columns=t)
data_kc.to_excel('results_kc.xlsx') #Save centroids
cluster_counts = pd.Series(y_kmeans).value_counts().sort_index()
print("Number of samples in each cluster:")
print(cluster_counts)
```
