# Supplementary material for: Exploring Self-Management–Based Mobile Health User Typologies and Associations Between User Types and Satisfaction With Key Mobile Health Functions: Comparative Study of Various Fitness and Weight Management App User Types
Source: JMIR Med Inform. 2026 Feb 10;14:e64860. doi: 10.2196/64860 (PMC12933165; doi:10.2196/64860)
Supplement: Multimedia Appendix 4 [file medinform_v14i1e64860_app4.pdf]

STROBE Statement—checklist of items that should be included in reports of observational studies

	Item No.	Recommendation	Page No.	Relevant text from manuscript
**Title and abstract**	1	(*a*) Indicate the study's design with a commonly used term in the title or the abstract	2-3	See *Abstract*
		(*b*) Provide in the abstract an informative and balanced summary of what was done and what was found	2-3	See *Abstract*
**Introduction**				
Background/rationale	2	Explain the scientific background and rationale for the investigation being reported	4-6	See *1. Introduction*
Objectives	3	State specific objectives, including any prespecified hypotheses	6	"Research question 1: User typology. : How can users of fitness and weight management apps be systematically categorized using quantitative methods based on self-management characteristics? What are the defining attributes of each user type? Research question RQ2: Satisfaction differences. Do user groups with distinct self-management characteristics demonstrate statistically significant differences in satisfaction with various mHealth functional designs? What are the specific manifestations of these differences, and which factors are relevant?"
**Methods**				
Study design	4	Present key elements of study design early in the paper	6	"Guided by self-management theory, this study utilizes the HBM and BCW to select variables representing the self-management characteristics of mHealth users. Data were collected through a questionnaire survey, and unsupervised clustering analysis was used to identify user clusters. Subsequently, the characteristic distributions of different user clusters were analyzed to construct user profile. Through inter-group difference analysis, this study compared the varying satisfaction levels of different user types with the various functions design of mHealth, and analyzed the correlation between user characteristics and satisfaction of functional designs. The entire workflow is illustrated in Figure 1."
Setting	5	Describe the setting, locations, and relevant dates, including periods of recruitment, exposure, follow-	13-14	"The survey was directed towards individuals who had engaged with exercise, fitness, or weight loss applications within the preceding three months.

		up, and data collection		During the period from June 28, 2022, to September 8, 2022, a snowball sampling survey was carried out via the WeChat platform. This was accomplished by disseminating a questionnaire link, which was generated using Wenjuanxing, in Moments and various group chats.”
Participants	6	(*a*) *Cohort study*—Give the eligibility criteria, and the sources and methods of selection of participants. Describe methods of follow-up<br>*Case-control study*—Give the eligibility criteria, and the sources and methods of case ascertainment and control selection. Give the rationale for the choice of cases and controls<br>*Cross-sectional study*—Give the eligibility criteria, and the sources and methods of selection of participants	13-14	“The survey was directed towards individuals who had engaged with exercise, fitness, or weight loss applications within the preceding three months.”<br>“A cumulative total of 2518 questionnaires were amassed. Among these, 902 participants indicated that they had not utilized the target applications for a three-month duration. Additionally, 591 responses were deemed invalid due to reasons such as incomplete answers, abbreviated response times, or the inability to pass quality control questions. Consequently, 1025 questionnaires remained suitable for subsequent analysis.”
		(*b*) *Cohort study*—For matched studies, give matching criteria and number of exposed and unexposed<br>*Case-control study*—For matched studies, give matching criteria and the number of controls per case		Not Applicable
Variables	7	Clearly define all outcomes, exposures, predictors, potential confounders, and effect modifiers. Give diagnostic criteria, if applicable	12-13	“Based on the constructed User Health Management Characteristics Framework, this study involves the measurement of nine independent variables along with five dependent variables assessing satisfaction with mHealth functionalities.”
Data sources/ measurement	8*	For each variable of interest, give sources of data and details of methods of assessment (measurement). Describe comparability of assessment methods if there is more than one group	13	“Specifically, the User Health Management Characteristics Framework includes four variables from the Health Belief Model (Perceived severity, Perceived susceptibility, Perceived barrier, Perceived benefit), with measurement items adapted from HBM-related studies by Ahadzadeh[53], Saghafi-Asl[36], McArthur[54], and Saghafi-Asl[36]; three variables from the Behavior Change Wheel (eHealth literacy, Perceived health status, Health literacy), measured using items were drawn from Behavior Change Wheel studies[55–57] and the Brief Health Literacy Screen (BHLS) scale[58]; and two variables combining both models (Health self-efficacy, Health

				management intention), with measurement items adapted from health management studies, such as Zhou[59] and Li[60]. For the satisfaction measurement of the five mHealth functional designs, this study first introduced the definition and specific design examples of each function in the questionnaire, followed by item measurement. The satisfaction measurement items were adapted from relevant studies on mHealth satisfaction[61].<br>The measurement instrument adopted a 5-point Likert scale, with the range spanning from 1 (strongly disagree) to 5 (strongly agree). To enhance the validity and reliability of the scale, a pre-test was conducted involving 24 participants. Subsequently, based on their comprehensive evaluations and constructive suggestions, the scale underwent a series of revisions, ultimately culminating in the formation of the final questionnaire (see Multimedia Appendix 1. Measurement instrument)."
Bias	9	Describe any efforts to address potential sources of bias	15	"The data gathered for the study were analyzed so researchers may search for indications of bias from non-response. To accomplish this and check for non-response bias[11], The Mann-Whitney U test was used to compare the responses of the first 20% and the last 20% of the data. According to the data, the two groups had no significant differences in the Health Guidance(Z=-0.728, P=0.466), Health Education(Z=-1.171, P=0.242), Health Monitoring(Z=-0.857, P=0.392), Social function(Z=-1.794, P=0.073), Gamification(Z=-0.260, P=0.795) constructs. Therefore, there was no problem with bias in this study caused by non-respondents not participating."
Study size	10	Explain how the study size was arrived at	14	"In accordance with the N:q Hypothesis[62], the ratio of sample size to item parameters within the scale was required to fall within the range of 20:1 to 10:1. In this particular study, as 51 items were designated for measurement, adhering to the more stringent sample ratio of 20:1, the requisite sample size was approximately 1020."
Quantitative variables	11	Explain how quantitative variables were handled in the analyses. If applicable, describe which groupings were chosen and why	14-15	"To address the question "What are the common combinations of health management characteristics in users during the process of using mHealth?",……we conducted a partial least squares analysis and compared the impact of health management characteristics on user satisfaction across different user groups in SmartPLS 3. The

				significance level for the tests was set at *P*< .05 for two-tailed tests. ”
Statistical methods	12	(*a*) Describe all statistical methods, including those used to control for confounding	18,19 22,24	“cluster analysis was performed on the sample by employing the Z-score standardized outcomes of nine health management indicators.”<br>“We further analyzed the differences in each indicator across the six clusters using the Kruskal-Wallis test to determine the uniqueness of each cluster.”<br>“the Kruskal-Wallis test was used to analyze the differences in satisfaction with the five functional designs of the mobile health app among all users.”<br>“Using nine health management characteristics as independent variables and the satisfaction with five feature designs as dependent variables, partial least squares (PLS) analysis was conducted to examine the impact of health management characteristics on the satisfaction with the functional design of mobile health apps.”<br>“The R2 value represents the proportion of the variance in the dependent variable that can be explained by the independent variables and is used to interpret the goodness-of-fit of the regression equation, while β indicates the correlation between variables.”
		(*b*) Describe any methods used to examine subgroups and interactions	21	“Firstly, the Kruskal-Wallis test was used to analyze the differences in satisfaction with the five functional designs of the mobile health app among all users.……It allows for a detailed examination of how each cluster differs from the others in terms of the satisfaction for specific functional designs.”
		(*c*) Explain how missing data were addressed	14	“Additionally, 591 responses were deemed invalid due to reasons such as incomplete answers, abbreviated response times, or the inability to pass quality control questions”
		(*d*) *Cohort study*—If applicable, explain how loss to follow-up was addressed<br>*Case-control study*—If applicable, explain how matching of cases and controls was addressed<br>*Cross-sectional study*—If applicable, describe analytical methods taking account of sampling strategy	14-15,17	“During the period from June 28, 2022, to September 8, 2022, a snowball sampling survey was carried out via the WeChat platform.”<br>“The data gathered for the study were analyzed so researchers may search for indications of bias from non-response. To accomplish this and check for non-response bias[11], The Mann-Whitney U test was used to compare the responses of the first 20% and the last 20% of the data. According to the data, the two groups had no significant differences in the Health Guidance(Z=-0.728, P=0.466), Health Education(Z=-1.171, P=0.242), Health Monitoring(Z=-0.857, P=0.392), Social function(Z=-1.794, P=0.073), Gamification(Z=-0.260, P=0.795) constructs. Therefore, there was no problem with bias in this study caused by non-respondents not participating.”

				"we found the distribution of the average values for each variable was not normally distributed, therefore, we used medians and interquartile ranges to represent as shown in Table 2."
		(*e*) Describe any sensitivity analyses	17	"The Elbow Method identifies the inflection point by analyzing the relationship between the within-cluster sum of squared errors (SSE) and the number of clusters, while the Silhouette Coefficient is used to mathematically assess the compactness and separation of the clustering structure. The results showed that when the number of clusters was six6, an inflection point appeared in the SSE curve, and the Silhouette Coefficient reached a locally optimal value of 0.1514 (see Multimedia Appendix 2), indicating that this clustering solution maintains good within-cluster compactness while achieving favorable between-cluster separation.)."
**Results**				
Participants	13*	(a) Report numbers of individuals at each stage of study—eg numbers potentially eligible, examined for eligibility, confirmed eligible, included in the study, completing follow-up, and analysed	14	"A cumulative total of 2518 questionnaires were amassed. Among these, 902 participants indicated that they had not utilized the target applications for a three-month duration. Additionally, 591 responses were deemed invalid due to reasons such as incomplete answers, abbreviated response times, or the inability to pass quality control questions."
		(b) Give reasons for non-participation at each stage	14	"Additionally, 591 responses were deemed invalid due to reasons such as incomplete answers, abbreviated response times, or the inability to pass quality control questions."
		(c) Consider use of a flow diagram		Not Applicable
Descriptive data	14*	(a) Give characteristics of study participants (eg demographic, clinical, social) and information on exposures and potential confounders	18	see Table 3. Demographic information(n=1025)
		(b) Indicate number of participants with missing data for each variable of interest		Not Applicable
		(c) *Cohort study*—Summarise follow-up time (eg, average and total amount)		Not Applicable
Outcome data	15*	*Cohort study*—Report numbers of outcome events or summary measures over time		Not Applicable
		Case-control study—Report numbers in each		Not Applicable

		exposure category, or summary measures of exposure		
		Cross-sectional study—Report numbers of outcome events or summary measures	17-18	See Table 5. Descriptive analysis of health management characteristics and satisfaction
Main results	16	(*a*) Give unadjusted estimates and, if applicable, confounder-adjusted estimates and their precision (eg, 95% confidence interval). Make clear which confounders were adjusted for and why they were included	22	Table 6. Cluster variables Z-Scores for the six-cluster solution.<br>Table 7. Median and difference of six user types' satisfaction with different characteristics.
		(*b*) Report category boundaries when continuous variables were categorized		Not Applicable, no category of continuous variables
		(*c*) If relevant, consider translating estimates of relative risk into absolute risk for a meaningful time period		Not Applicable
Other analyses	17	Report other analyses done—eg analyses of subgroups and interactions, and sensitivity analyses		Not Applicable
**Discussion**				
Key results	18	Summarise key results with reference to study objectives	14-23	See the first four paragraphs of the discussion
Limitations	19	Discuss limitations of the study, taking into account sources of potential bias or imprecision. Discuss both direction and magnitude of any potential bias	28	See the fifth paragraphs of the discussion
Interpretation	20	Give a cautious overall interpretation of results considering objectives, limitations, multiplicity of analyses, results from similar studies, and other relevant evidence	24-28	“This study categorizes mHealth users into six distinct groups based on their self-management characteristics. It reveals a complex classification derived from self-management-related attributes such as knowledge, beliefs, self-regulation skills and abilities, and motivation….”
Generalisability	21	Discuss the generalisability (external validity) of the study results	28	“The findings of this study offer clear implications for the personalized design and practice of mHealth. Mobile health developers should adopt a user-centered design concept and, based on the user types constructed in this study, tailor functional modules accordingly.”

**Other information**				
Funding	22	Give the source of funding and the role of the funders for the present study and, if applicable, for the original study on which the present article is based	31	“This work was supported by the National Natural Science Foundation of China [Grant 72404112, 72574006]….”

*Give information separately for cases and controls in case-control studies and, if applicable, for exposed and unexposed groups in cohort and cross-sectional studies.

Note: An Explanation and Elaboration article discusses each checklist item and gives methodological background and published examples of transparent reporting. The STROBE checklist is best used in conjunction with this article (freely available on the Web sites of PLoS Medicine at http://www.plosmedicine.org/, Annals of Internal Medicine at http://www.annals.org/, and Epidemiology at http://www.epidem.com/). Information on the STROBE Initiative is available at www.strobe-statement.org.
