# Supplementary material for: Exploring Self-Management–Based Mobile Health User Typologies and Associations Between User Types and Satisfaction With Key Mobile Health Functions: Comparative Study of Various Fitness and Weight Management App User Types
Source: JMIR Med Inform. 2026 Feb 10;14:e64860. doi: 10.2196/64860 (PMC12933165; doi:10.2196/64860)
Supplement: Multimedia Appendix 5 [file medinform_v14i1e64860_app5.pdf]

Hypothesis Test Summary

	Null Hypothesis	Test	Sig.	Decision
1	The distribution of Perceived severity is the same across categories of cluster.	Independent-Samples Kruskal-Wallis Test	.000	Reject the null hypothesis.
2	The distribution of Perceived susceptibility is the same across categories of cluster.	Independent-Samples Kruskal-Wallis Test	.000	Reject the null hypothesis.
3	The distribution of Perceived barrier is the same across categories of cluster.	Independent-Samples Kruskal-Wallis Test	.000	Reject the null hypothesis.
4	The distribution of Perceived benefit is the same across categories of cluster.	Independent-Samples Kruskal-Wallis Test	.000	Reject the null hypothesis.
5	The distribution of Perceived health status is the same across categories of cluster.	Independent-Samples Kruskal-Wallis Test	.000	Reject the null hypothesis.
6	The distribution of eHealth literacy is the same across categories of cluster.	Independent-Samples Kruskal-Wallis Test	.000	Reject the null hypothesis.
7	The distribution of health self-efficacy is the same across categories of cluster.	Independent-Samples Kruskal-Wallis Test	.000	Reject the null hypothesis.
8	The distribution of health management intention is the same across categories of cluster.	Independent-Samples Kruskal-Wallis Test	.000	Reject the null hypothesis.

Asymptotic significances are displayed. The significance level is .050.

Independent-Samples Kruskal-Wallis Test

Perceived severity across cluster

Pairwise Comparisons of cluster

Sample 1-Sample 2	Test Statistic	Std. Error	Std. Test Statistic	Sig.	Adj. Sig.[a]
6-4	92.179	30.185	3.054	.002	.034
6-2	353.700	33.924	10.426	.000	.000
6-5	368.946	31.519	11.706	.000	.000
6-1	444.251	33.591	13.225	.000	.000
6-3	481.429	31.160	15.450	.000	.000
4-2	261.520	31.926	8.191	.000	.000
4-5	-276.767	29.358	-9.427	.000	.000
4-1	352.071	31.572	11.151	.000	.000
4-3	389.250	28.973	13.435	.000	.000
2-5	-15.246	33.190	-.459	.646	1.000
2-1	90.551	35.163	2.575	.010	.150
2-3	-127.730	32.850	-3.888	.000	.002
5-1	75.305	32.850	2.292	.022	.328
5-3	112.483	30.360	3.705	.000	.003
1-3	-37.179	32.506	-1.144	.253	1.000

Each row tests the null hypothesis that the Sample 1 and Sample 2 distributions are the same.

Asymptotic significances (2-sided tests) are displayed. The significance level is .05.

a. Significance values have been adjusted by the Bonferroni correction for multiple tests.

Perceived susceptibility across cluster

Pairwise Comparisons of cluster

Sample 1-Sample 2	Test Statistic	Std. Error	Std. Test Statistic	Sig.	Adj. Sig.[a]
1-4	-134.560	31.712	-4.243	.000	.000
1-6	-269.533	33.740	-7.989	.000	.000
1-2	-512.242	35.319	-14.503	.000	.000
1-5	-529.256	32.995	-16.040	.000	.000
1-3	-548.216	32.650	-16.791	.000	.000
4-6	-134.973	30.319	-4.452	.000	.000
4-2	377.682	32.068	11.778	.000	.000
4-5	-394.696	29.489	-13.385	.000	.000
4-3	413.656	29.102	14.214	.000	.000
6-2	242.708	34.074	7.123	.000	.000
6-5	259.722	31.659	8.204	.000	.000
6-3	278.682	31.299	8.904	.000	.000
2-5	-17.014	33.337	-.510	.610	1.000
2-3	-35.974	32.995	-1.090	.276	1.000
5-3	18.960	30.495	.622	.534	1.000

Each row tests the null hypothesis that the Sample 1 and Sample 2 distributions are the same.

Asymptotic significances (2-sided tests) are displayed. The significance level is .05.

a. Significance values have been adjusted by the Bonferroni correction for multiple tests.

Perceived barrier across cluster

Pairwise Comparisons of cluster

Sample 1-Sample 2	Test Statistic	Std. Error	Std. Test Statistic	Sig.	Adj. Sig.[a]
1-3	-71.032	32.668	-2.174	.030	.445
1-4	-194.086	31.730	-6.117	.000	.000
1-6	-469.812	33.758	-13.917	.000	.000
1-5	-530.101	33.013	-16.057	.000	.000
1-2	-672.999	35.339	-19.044	.000	.000
3-4	-123.054	29.117	-4.226	.000	.000
3-6	-398.780	31.316	-12.734	.000	.000
3-5	-459.069	30.511	-15.046	.000	.000
3-2	601.967	33.013	18.234	.000	.000
4-6	-275.726	30.336	-9.089	.000	.000
4-5	-336.015	29.505	-11.388	.000	.000
4-2	478.913	32.085	14.926	.000	.000
6-5	60.289	31.676	1.903	.057	.855
6-2	203.188	34.093	5.960	.000	.000
5-2	142.898	33.356	4.284	.000	.000

Each row tests the null hypothesis that the Sample 1 and Sample 2 distributions are the same.

Asymptotic significances (2-sided tests) are displayed. The significance level is .05.

a. Significance values have been adjusted by the Bonferroni correction for multiple tests.

Perceived benefit across cluster

Pairwise Comparisons of cluster

Sample 1-Sample 2	Test Statistic	Std. Error	Std. Test Statistic	Sig.	Adj. Sig.[a]
6-4	311.311	29.349	10.607	.000	.000
6-2	350.132	32.984	10.615	.000	.000
6-5	447.376	30.646	14.598	.000	.000
6-3	579.309	30.297	19.121	.000	.000
6-1	596.544	32.660	18.265	.000	.000
4-2	38.821	31.042	1.251	.211	1.000
4-5	-136.065	28.545	-4.767	.000	.000
4-3	267.998	28.170	9.514	.000	.000
4-1	285.233	30.697	9.292	.000	.000
2-5	-97.244	32.270	-3.013	.003	.039
2-3	-229.177	31.939	-7.175	.000	.000
2-1	246.413	34.189	7.207	.000	.000
5-3	131.933	29.519	4.469	.000	.000
5-1	149.168	31.939	4.670	.000	.000
3-1	17.235	31.605	.545	.586	1.000

Each row tests the null hypothesis that the Sample 1 and Sample 2 distributions are the same.

Asymptotic significances (2-sided tests) are displayed. The significance level is .05.

a. Significance values have been adjusted by the Bonferroni correction for multiple tests.

Perceived health status across cluster

Pairwise Comparisons of cluster

Sample 1-Sample 2	Test Statistic	Std. Error	Std. Test Statistic	Sig.	Adj. Sig.[a]
5-6	-167.042	31.762	-5.259	.000	.000
5-3	306.947	30.594	10.033	.000	.000
5-4	375.050	29.585	12.677	.000	.000
5-2	426.979	33.446	12.766	.000	.000
5-1	677.634	33.103	20.471	.000	.000
6-3	139.905	31.400	4.456	.000	.000
6-4	208.008	30.418	6.838	.000	.000
6-2	259.937	34.185	7.604	.000	.000
6-1	510.592	33.850	15.084	.000	.000
3-4	-68.103	29.196	-2.333	.020	.295
3-2	120.032	33.103	3.626	.000	.004
3-1	370.687	32.756	11.317	.000	.000
4-2	51.929	32.172	1.614	.107	1.000
4-1	302.584	31.816	9.511	.000	.000
2-1	250.655	35.434	7.074	.000	.000

Each row tests the null hypothesis that the Sample 1 and Sample 2 distributions are the same.

Asymptotic significances (2-sided tests) are displayed. The significance level is .05.

a. Significance values have been adjusted by the Bonferroni correction for multiple tests.

eHealth literacy across cluster

Pairwise Comparisons of cluster

Sample 1-Sample 2	Test Statistic	Std. Error	Std. Test Statistic	Sig.	Adj. Sig.[a]
6-5	58.117	31.858	1.824	.068	1.000
6-4	192.435	30.510	6.307	.000	.000
6-2	331.060	34.288	9.655	.000	.000
6-3	434.343	31.495	13.791	.000	.000
6-1	470.098	33.952	13.846	.000	.000
5-4	134.318	29.674	4.526	.000	.000
5-2	272.943	33.547	8.136	.000	.000
5-3	376.226	30.686	12.260	.000	.000
5-1	411.981	33.202	12.408	.000	.000
4-2	138.625	32.269	4.296	.000	.000
4-3	241.908	29.284	8.261	.000	.000
4-1	277.663	31.911	8.701	.000	.000
2-3	-103.284	33.202	-3.111	.002	.028
2-1	139.038	35.541	3.912	.000	.001
3-1	35.755	32.855	1.088	.276	1.000

Each row tests the null hypothesis that the Sample 1 and Sample 2 distributions are the same.

Asymptotic significances (2-sided tests) are displayed. The significance level is .05.

a. Significance values have been adjusted by the Bonferroni correction for multiple tests.

Health management intention across cluster

Pairwise Comparisons of cluster

Sample 1-Sample 2	Test Statistic	Std. Error	Std. Test Statistic	Sig.	Adj. Sig.[a]
6-5	161.813	31.564	5.126	.000	.000
6-4	183.238	30.228	6.062	.000	.000
6-2	404.710	33.972	11.913	.000	.000
6-1	455.252	33.639	13.534	.000	.000
6-3	504.996	31.205	16.183	.000	.000
5-4	21.425	29.400	.729	.466	1.000
5-2	242.897	33.237	7.308	.000	.000
5-1	293.439	32.897	8.920	.000	.000
5-3	343.183	30.403	11.288	.000	.000
4-2	221.472	31.972	6.927	.000	.000
4-1	272.014	31.617	8.603	.000	.000
4-3	321.758	29.014	11.090	.000	.000
2-1	50.542	35.214	1.435	.151	1.000
2-3	-100.286	32.897	-3.049	.002	.034
1-3	-49.744	32.552	-1.528	.126	1.000

Each row tests the null hypothesis that the Sample 1 and Sample 2 distributions are the same.

Asymptotic significances (2-sided tests) are displayed. The significance level is .05.

a. Significance values have been adjusted by the Bonferroni correction for multiple tests.

health self-efficacy across cluster

Pairwise Comparisons of cluster

Sample 1-Sample 2	Test Statistic	Std. Error	Std. Test Statistic	Sig.	Adj. Sig.[a]
6-5	56.876	31.695	1.794	.073	1.000
6-4	279.399	30.353	9.205	.000	.000
6-2	395.925	34.113	11.606	.000	.000
6-3	481.938	31.334	15.381	.000	.000
6-1	589.339	33.778	17.448	.000	.000
5-4	222.523	29.522	7.538	.000	.000
5-2	339.049	33.375	10.159	.000	.000
5-3	425.063	30.529	13.923	.000	.000
5-1	532.463	33.033	16.119	.000	.000
4-2	116.526	32.104	3.630	.000	.004
4-3	202.540	29.134	6.952	.000	.000
4-1	309.940	31.748	9.762	.000	.000
2-3	-86.014	33.033	-2.604	.009	.138
2-1	193.414	35.359	5.470	.000	.000
3-1	107.400	32.687	3.286	.001	.015

Each row tests the null hypothesis that the Sample 1 and Sample 2 distributions are the same.

Asymptotic significances (2-sided tests) are displayed. The significance level is .05.

a. Significance values have been adjusted by the Bonferroni correction for multiple tests.
