# Supplementary material for: Exploring Self-Management–Based Mobile Health User Typologies and Associations Between User Types and Satisfaction With Key Mobile Health Functions: Comparative Study of Various Fitness and Weight Management App User Types
Source: JMIR Med Inform. 2026 Feb 10;14:e64860. doi: 10.2196/64860 (PMC12933165; doi:10.2196/64860)
Supplement: Multimedia Appendix 6 [file medinform_v14i1e64860_app6.pdf]

Hypothesis Test Summary

	Null Hypothesis	Test	Sig.	Decision
1	The distribution of satisfaction with Health Guidance is the same across categories of cluster.	Independent-Samples Kruskal-Wallis Test	.000	Reject the null hypothesis.
2	The distribution of satisfaction with Health Education is the same across categories of cluster.	Independent-Samples Kruskal-Wallis Test	.000	Reject the null hypothesis.
3	The distribution of satisfaction with Health Monitoring is the same across categories of cluster.	Independent-Samples Kruskal-Wallis Test	.000	Reject the null hypothesis.
4	The distribution of satisfaction with Social Function is the same across categories of cluster.	Independent-Samples Kruskal-Wallis Test	.000	Reject the null hypothesis.
5	The distribution of satisfaction with Gamification is the same across categories of cluster.	Independent-Samples Kruskal-Wallis Test	.000	Reject the null hypothesis.

Asymptotic significances are displayed. The significance level is .050.

Satisfaction with Health Guidance

Pairwise Comparisons of cluster

Sample 1-Sample 2	Test Statistic	Std. Error	Std. Test Statistic	Sig.	Adj. Sig.[a]
6-4	86.039	30.114	2.857	.004	.064
6-5	115.539	31.444	3.674	.000	.004
6-1	209.302	33.511	6.246	.000	.000
6-3	214.101	31.087	6.887	.000	.000
6-2	284.435	33.843	8.404	.000	.000
4-5	-29.500	29.289	-1.007	.314	1.000
4-1	123.264	31.497	3.913	.000	.001
4-3	128.062	28.904	4.431	.000	.000
4-2	198.397	31.851	6.229	.000	.000
5-1	93.763	32.772	2.861	.004	.063
5-3	98.562	30.288	3.254	.001	.017
5-2	168.896	33.111	5.101	.000	.000
1-3	-4.798	32.429	-.148	.882	1.000
1-2	-75.133	35.080	-2.142	.032	.483
3-2	70.334	32.772	2.146	.032	.478

Each row tests the null hypothesis that the Sample 1 and Sample 2 distributions are the same.
Asymptotic significances (2-sided tests) are displayed. The significance level is .05.
a. Significance values have been adjusted by the Bonferroni correction for multiple tests.

Satisfaction with Health Education

Pairwise Comparisons of cluster

Sample 1-Sample 2	Test Statistic	Std. Error	Std. Test Statistic	Sig.	Adj. Sig.[a]
6-5	86.956	31.443	2.765	.006	.085
6-4	94.952	30.113	3.153	.002	.024
6-3	202.659	31.086	6.519	.000	.000
6-1	220.841	33.510	6.590	.000	.000
6-2	269.163	33.842	7.953	.000	.000
5-4	7.996	29.288	.273	.785	1.000
5-3	115.703	30.287	3.820	.000	.002
5-1	133.885	32.771	4.085	.000	.001
5-2	182.206	33.110	5.503	.000	.000
4-3	107.707	28.903	3.726	.000	.003
4-1	125.888	31.496	3.997	.000	.001
4-2	174.210	31.850	5.470	.000	.000
3-1	18.181	32.428	.561	.575	1.000
3-2	66.503	32.771	2.029	.042	.636
1-2	-48.322	35.079	-1.378	.168	1.000

Each row tests the null hypothesis that the Sample 1 and Sample 2 distributions are the same.
Asymptotic significances (2-sided tests) are displayed. The significance level is .05.
a. Significance values have been adjusted by the Bonferroni correction for multiple tests.

Satisfaction with Health Monitoring

Pairwise Comparisons of cluster

Sample 1-Sample 2	Test Statistic	Std. Error	Std. Test Statistic	Sig.	Adj. Sig.[a]
6-4	65.173	30.089	2.166	.030	.455
6-5	129.395	31.418	4.118	.000	.001
6-2	230.225	33.815	6.808	.000	.000
6-3	244.704	31.061	7.878	.000	.000
6-1	249.018	33.483	7.437	.000	.000
4-5	-64.223	29.265	-2.195	.028	.423
4-2	165.052	31.824	5.186	.000	.000
4-3	179.531	28.880	6.216	.000	.000
4-1	183.846	31.471	5.842	.000	.000
5-2	100.830	33.084	3.048	.002	.035
5-3	115.308	30.263	3.810	.000	.002
5-1	119.623	32.745	3.653	.000	.004
2-3	-14.479	32.745	-.442	.658	1.000
2-1	18.793	35.051	.536	.592	1.000
3-1	4.315	32.402	.133	.894	1.000

Each row tests the null hypothesis that the Sample 1 and Sample 2 distributions are the same.
Asymptotic significances (2-sided tests) are displayed. The significance level is .05.
a. Significance values have been adjusted by the Bonferroni correction for multiple tests.

Satisfaction with Social Function

Pairwise Comparisons of cluster

Sample 1-Sample 2	Test Statistic	Std. Error	Std. Test Statistic	Sig.	Adj. Sig.[a]
5-4	26.423	29.379	.899	.368	1.000
5-6	-42.096	31.541	-1.335	.182	1.000
5-3	94.326	30.381	3.105	.002	.029
5-1	119.383	32.873	3.632	.000	.004
5-2	245.503	33.213	7.392	.000	.000
4-6	-15.673	30.206	-.519	.604	1.000
4-3	67.903	28.993	2.342	.019	.288
4-1	92.960	31.594	2.942	.003	.049
4-2	219.080	31.949	6.857	.000	.000
6-3	52.230	31.182	1.675	.094	1.000
6-1	77.287	33.614	2.299	.021	.322
6-2	203.407	33.947	5.992	.000	.000
3-1	25.057	32.528	.770	.441	1.000
3-2	151.177	32.873	4.599	.000	.000
1-2	-126.120	35.188	-3.584	.000	.005

Each row tests the null hypothesis that the Sample 1 and Sample 2 distributions are the same.
Asymptotic significances (2-sided tests) are displayed. The significance level is .05.
a. Significance values have been adjusted by the Bonferroni correction for multiple tests.

Satisfaction with Gamification

Pairwise Comparisons of cluster

Sample 1-Sample 2	Test Statistic	Std. Error	Std. Test Statistic	Sig.	Adj. Sig.[a]
5-6	-32.493	31.397	-1.035	.301	1.000
5-4	51.038	29.245	1.745	.081	1.000
5-3	73.942	30.242	2.445	.014	.217
5-1	97.977	32.722	2.994	.003	.041
5-2	238.051	33.061	7.200	.000	.000
6-4	18.546	30.068	.617	.537	1.000
6-3	41.449	31.040	1.335	.182	1.000
6-1	65.484	33.461	1.957	.050	.755
6-2	205.558	33.792	6.083	.000	.000
4-3	22.903	28.861	.794	.427	1.000
4-1	46.939	31.450	1.492	.136	1.000
4-2	187.012	31.803	5.880	.000	.000
3-1	24.035	32.380	.742	.458	1.000
3-2	164.109	32.722	5.015	.000	.000
1-2	-140.074	35.027	-3.999	.000	.001

Each row tests the null hypothesis that the Sample 1 and Sample 2 distributions are the same.
Asymptotic significances (2-sided tests) are displayed. The significance level is .05.
a. Significance values have been adjusted by the Bonferroni correction for multiple tests.
