# Supplementary material for: Exploring Self-Management–Based Mobile Health User Typologies and Associations Between User Types and Satisfaction With Key Mobile Health Functions: Comparative Study of Various Fitness and Weight Management App User Types
Source: JMIR Med Inform. 2026 Feb 10;14:e64860. doi: 10.2196/64860 (PMC12933165; doi:10.2196/64860)
Supplement: Multimedia Appendix 7 [file medinform_v14i1e64860_app7.pdf]

Table 1. Construct reliability and convergent validity.

Construct and items	Factor loadings	Composite reliability	Average variance extracted	Cronbach alphas
Satisfaction with Gamification_1	0.885	0.937	0.831	0.899
Satisfaction with Gamification_2	0.936			
Satisfaction with Gamification_3	0.914			
Satisfaction with Health Education_1	0.881	0.924	0.802	0.877
Satisfaction with Health Education_2	0.905			
Satisfaction with Health Education_3	0.901			
Satisfaction with Health Guidance_1	0.887	0.934	0.826	0.895
Satisfaction with Health Guidance_2	0.922			
Satisfaction with Health Guidance_3	0.917			
Satisfaction with Health Monitoring_1	0.868	0.909	0.770	0.851
Satisfaction with Health Monitoring_2	0.887			
Satisfaction with Health Monitoring_3	0.877			
Health management intention_1	0.853	0.861	0.674	0.757
Health management intention_2	0.861			
Health management intention_3	0.744			
Health self-efficacy_1	0.800	0.861	0.673	0.758
Health self-efficacy_2	0.830			
Health self-efficacy_3	0.831			
Perceived barrier_1	0.846	0.875	0.778	0.720
Perceived barrier_2	0.917			
Perceived benefit_1	0.847	0.918	0.737	0.881
Perceived benefit_2	0.869			
Perceived benefit_3	0.867			

Perceived benefit_4	0.851			
Perceived health status_1	0.862	0.921	0.795	0.871
Perceived health status_2	0.900			
Perceived health status_3	0.912			
Perceived severity_1	0.891	0.878	0.644	0.820
Perceived severity_2	0.871			
Perceived severity_3	0.718			
Perceived severity_4	0.714			
Perceived susceptibility_1	0.844	0.868	0.767	0.700
Perceived susceptibility_2	0.907			
Satisfaction with Social Function_1	0.889	0.928	0.811	0.884
Satisfaction with Social Function_2	0.912			
Satisfaction with Social Function_3	0.901			
eHealth literacy_1	0.788	0.954	0.721	0.945
eHealth literacy_2	0.847			
eHealth literacy_3	0.875			
eHealth literacy_4	0.891			
eHealth literacy_5	0.883			
eHealth literacy_6	0.845			
eHealth literacy_7	0.837			
eHealth literacy_8	0.821			

Table 2. Discriminant Validity - Heterotrait-Monotrait Ratio (HTMT) Matrix

	S-G	S-HE	S-HG	S-HM	HM	HS	PB	PBE	PH	PSE	PSU	S-S	eH
**S-G**													
**S-HE**	0.468												
**S-HG**	0.439	0.832											
**S-HM**	0.355	0.687	0.711										
**HM**	0.178	0.382	0.370	0.319									
**HS**	0.193	0.368	0.355	0.301	0.680								
**PB**	0.068	0.038	0.029	0.057	0.343	0.489							
**PBE**	0.041	0.213	0.253	0.287	0.491	0.532	0.259						
**PH**	0.163	0.254	0.226	0.203	0.322	0.610	0.387	0.208					
**PSE**	0.064	0.207	0.239	0.298	0.435	0.294	0.103	0.519	0.085				
**PSU**	0.106	0.084	0.134	0.103	0.136	0.100	0.423	0.087	0.487	0.334			
**S-S**	0.819	0.603	0.517	0.423	0.264	0.285	0.045	0.028	0.216	0.100	0.111		
**eH**	0.155	0.269	0.279	0.301	0.408	0.412	0.385	0.314	0.350	0.281	0.035	0.191	

⁎ S-G: Satisfaction with Gamification, S-HE: Satisfaction with Health Education, S-HG: Satisfaction with Health Guidance, S-HM: Satisfaction with Health Monitoring, HM: Health management intention, HS: Health self-efficacy, PB: Perceived barrier, PBE: Perceived benefit, PH: Perceived health status, PSE: Perceived severity, PSU: Perceived susceptibility, S-S: Satisfaction with Social Function, eH: eHealth literacy
