# Supplementary material for: Exploring Self-Management–Based Mobile Health User Typologies and Associations Between User Types and Satisfaction With Key Mobile Health Functions: Comparative Study of Various Fitness and Weight Management App User Types
Source: JMIR Med Inform. 2026 Feb 10;14:e64860. doi: 10.2196/64860 (PMC12933165; doi:10.2196/64860)
Supplement: Multimedia Appendix 8 [file medinform_v14i1e64860_app8.pdf]

Multimedia Appendix 8. Table 1. Path coefficients- Mean, STDEV, T value, p value

	Original sample (O)	Sample mean (M)	Standard deviation (STDEV)	T statistics (|O/STDEV|)	P values
**eHealth literacy -> Gamification**	0.119	0.118	0.043	2.771	0.006
**eHealth literacy -> Health Education**	0.131	0.128	0.039	3.362	0.001
**eHealth literacy -> Health Guidance**	0.153	0.149	0.039	3.947	0.000
**eHealth literacy -> Health Monitoring**	0.170	0.169	0.037	4.561	0.000
**eHealth literacy -> Social Function**	0.118	0.116	0.042	2.805	0.005
**Health management intention -> Gamification**	0.100	0.099	0.043	2.354	0.019
**Health management intention -> Health Education**	0.175	0.173	0.037	4.670	0.000
**Health management intention -> Health Guidance**	0.148	0.146	0.037	3.966	0.000
**Health management intention -> Health Monitoring**	0.083	0.081	0.040	2.064	0.039
**Health management intention -> Social Function**	0.139	0.138	0.041	3.396	0.001
**Health self-efficacy -> Gamification**	0.137	0.136	0.044	3.076	0.002
**Health self-efficacy -> Health Education**	0.147	0.144	0.042	3.491	0.000
**Health self-efficacy -> Health Guidance**	0.140	0.137	0.042	3.307	0.001
**Health self-efficacy -> Health Monitoring**	0.070	0.066	0.044	1.616	0.106
**Health self-efficacy -> Social Function**	0.195	0.192	0.042	4.598	0.000
**Perceived barrier -> Gamification**	0.139	0.136	0.045	3.074	0.002
**Perceived barrier -> Health Education**	0.143	0.132	0.046	3.130	0.002
**Perceived barrier -> Health Guidance**	0.177	0.164	0.049	3.586	0.000

**Perceived barrier -> Health Monitoring**	0.107	0.094	0.049	2.194	0.028
**Perceived barrier -> Social Function**	0.150	0.142	0.050	3.032	0.002
**Perceived benefit -> Gamification**	-0.165	-0.168	0.033	5.011	0.000
**Perceived benefit -> Health Education**	-0.001	-0.002	0.034	0.035	0.972
**Perceived benefit -> Health Guidance**	0.048	0.048	0.033	1.446	0.148
**Perceived benefit -> Health Monitoring**	0.089	0.089	0.037	2.428	0.015
**Perceived benefit -> Social Function**	-0.210	-0.212	0.036	5.906	0.000
**Perceived health status -> Gamification**	0.122	0.125	0.043	2.834	0.005
**Perceived health status -> Health Education**	0.120	0.122	0.039	3.072	0.002
**Perceived health status -> Health Guidance**	0.111	0.112	0.037	2.967	0.003
**Perceived health status -> Health Monitoring**	0.088	0.089	0.040	2.188	0.029
**Perceived health status -> Social Function**	0.135	0.138	0.040	3.391	0.001
**Perceived severity -> Gamification**	-0.012	-0.011	0.034	0.339	0.735
**Perceived severity -> Health Education**	0.050	0.051	0.033	1.520	0.128
**Perceived severity -> Health Guidance**	0.058	0.059	0.033	1.781	0.075
**Perceived severity -> Health Monitoring**	0.118	0.119	0.036	3.235	0.001
**Perceived severity -> Social Function**	0.034	0.035	0.034	0.995	0.320
**Perceived susceptibility -> Gamification**	0.099	0.102	0.045	2.200	0.028
**Perceived susceptibility -> Health Education**	0.049	0.056	0.038	1.282	0.200
**Perceived susceptibility -> Health Guidance**	0.076	0.084	0.037	2.056	0.040
**Perceived susceptibility -> Health Monitoring**	0.046	0.053	0.038	1.217	0.224
**Perceived susceptibility -> Social Function**	0.098	0.103	0.042	2.314	0.021
